# The effect of the neutral cytidine protonated analogue pseudoisocytidine on the stability of i-motif structures

**DOI:** 10.1038/s41598-017-02723-y

**Published:** 2017-06-05

**Authors:** B. Mir, X. Solés, C. González, N. Escaja

**Affiliations:** 1 Inorganic and Organic Chemistry Department, Organic Chemistry Section, and IBUB, University of Barcelona, Martí i Franquès 1-11, 08028 Barcelona, Spain; 2 Instituto de Química Física Rocasolano, CSIC, Serrano 119, 28006 Madrid, Spain; 3 BIOESTRAN, associated unit UB-CSIC, Spain

## Abstract

Incorporation of pseudoisocytidine (psC), a neutral analogue of protonated cytidine, in i-motifs has been studied by spectroscopic methods. Our results show that neutral psC:C base pairs can stabilize i-motifs at neutral pH, but the stabilization only occurs when psC:C base pairs are located at the ends of intercalated C:C^+^ stacks. When psC occupies central positions, the resulting i-motifs are only observed at low pH and psC:C^+^ or psC:psC^+^ hemiprotonated base pairs are formed instead of their neutral analogs. Overall, our results suggest that positively charged base pairs are necessary to stabilize this non-canonical DNA structure.

## Introduction

I-motifs are quadruplex structures formed by the intercalation of hemiprotonated C:C^+^ base pairs. They are usually found in repetitive C-rich sequences and, as protonation of cytosines is required, are more stable at acidic pH^1, 2^. Although these structures were first observed in 1993^3^, it has been during the last decade that they have attracted increased interest. In addition to their potential use in nanotechnology^4, 5^ and medical applications^6^, there is evidence of their possible role in biological processes, such as telomeric^7^ and centromeric formation^8, 9^ and as transcriptional regulators^10, 11^.

Stability of i-motif structures depends on the number of stacked C:C^+^ base pairs, and also on additional interactions between residues surrounding the C:C^+^ tracts^12, 13^. A particular case of stabilizing capping elements in i-motif structures are the minor groove tetrads G:T:G:T^14, 15^. These tetrads can provide enough stabilization to allow the formation of i-motif-like structures in sequences with very few C:C^+^ base pairs^15^.

Only a few examples of i-motif structures have been observed at neutral or nearly neutral pH^16–18^. However, i-motifs might exist in the cell since their formation appears to be favoured by negative super helicity^19^ or molecular crowded conditions^20^. The possibility of increasing the stability of the i-motif at physiological conditions has attracted much attention in recent years. With this aim, a number of chemical modifications have been tested by different groups, but most of them have led to destabilization^21, 22^. The exceptions are LNA and PNA for some selected sequences^23, 24^ and, especially, the 2′-F-araC substitution, which affords dramatic stabilization over a wide range of pH and nucleotidic sequences^25^.

**Figure 1:**
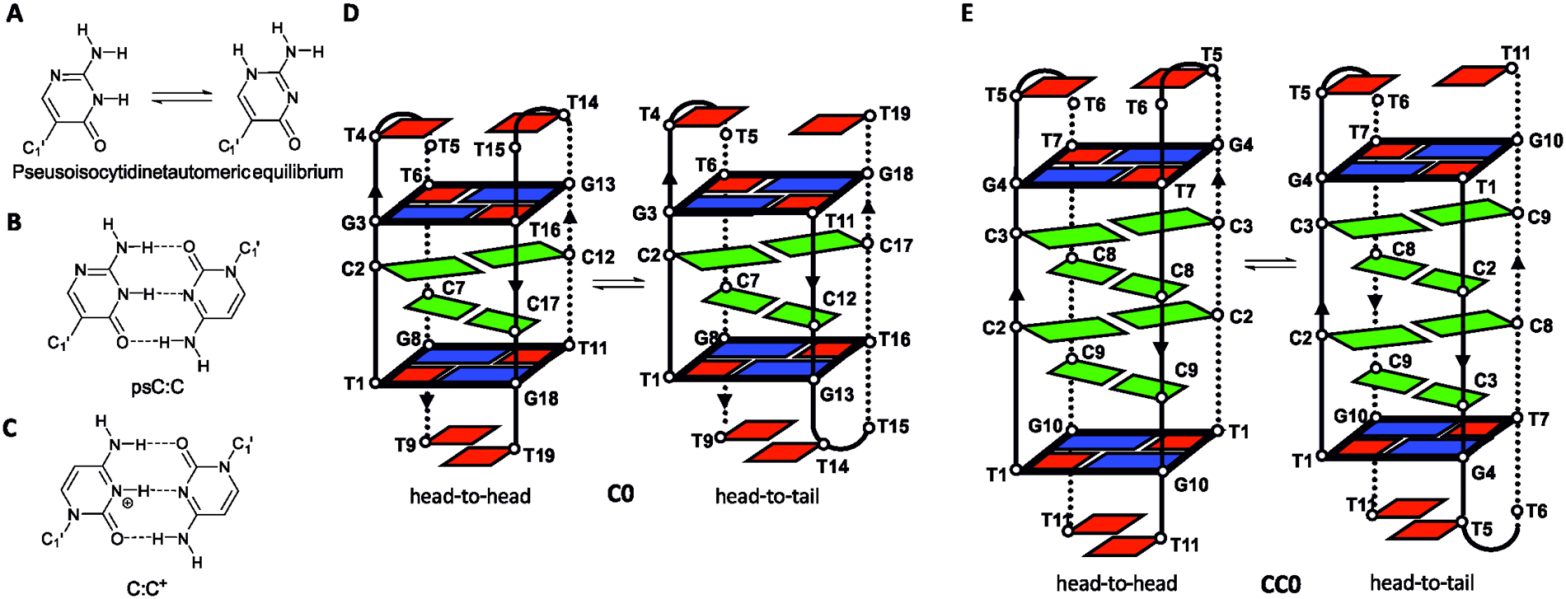
Expected base-pairs in psC-containing i-motifs and schematic representations of the structural equilibrium in **C0** and **CC0**. (**A**) Tautomeric equilibrium of pseudoisocytidine. (**B**) Neutral psC:C base pair. (**C**) Hemiprotonated C:C^+^ base pair in i-motif structures. (**D**,**E**) Head-to-head and head-to-tail dimeric i-motif structures of **C0** and **CC0**, respectively.

Neutral analogues of protonated cytidines may further increase the range of conditions in which i-motifs exist. In this paper, we explore the incorporation of pseudoisocytidine in i-motif forming sequences. Pseudoisocytidine is an isostere of cytidine with lower p*K*_a_ values^26^ (3.79 (N1) and 3.69 (N3)) that exhibits a tautomeric equilibrium providing an additional hydrogen-bond donor at the N3 position (see Fig. 1A). This modification has been used in previous studies to stabilize parallel triplexes by substituting protonated cytidine residues in C^+^:G:C triplets^27–30^. The appropriate pseudoisocytosine tautomer can hybridize with cytosine forming a neutral psC:C base pair (see Fig. 1B). This pair is isomorphic to the hemiprotonated C:C^+^ base pair and may yield more stable i-motifs at neutral conditions. In addition, studying the effect of neutral analogues on hemiprotonated C:C^+^ base pairs may help assess the role of charge compensation on the stability of i-motif structures.

Among the many families of known i-motifs, we decided to incorporate pseudoisocytidine in the mini i-motif structures cited above^15^. These structures are simple and relatively stable at pH close to 7, and are good models to study the effect of chemical modifications in i-motifs at neutral pH conditions. In particular, we have chosen the sequence d(TCGTTTCGT) (**C0**)^15^ and a longer analogue with an extended C:C^+^ tract, d(TCCGTTTCCGT) (**CC0**) (see Section 1 and Figs 1–4 in Supplementary Information for NMR characterization of **CC0**). These two sequences fold into dimeric i-motif structures formed by two or four C:C^+^ base pairs capped by two minor groove G:T:G:T tetrads (see Fig. 1D and E). In both cases, the two possible orientations between each subunit, head-to-head and head-to-tail, coexist in equilibrium. The effect of single psC substitutions on this equilibrium allows the simultaneous evaluation of psC replacements in different local environments within the i-motif. With this aim, we have studied three modified sequences. Two sequences, d(TCGTTT**psC**GT) (**C7**) and d(TCCGTTT**psC**CGT) (**CC8**), in which psC residues are located in the core of the structure (central C:C^+^ base pairs, positions 7 and 8 of **C0** and **CC0**, respectively), and one sequence, d(TCCGTTTC**psC**GT) (**CC9**), in which psC residues are located in the outer C:C^+^ base pairs (position 9 of **CC0**). In the modified sequences, the relative stability between the different i-motif species will be conditioned by the relative preference of forming C:C^+^, psC:C or psC:psC base pairs, the last one between the two tautomeric forms of psC (see Figure S5 in ESI). Formation of psC:C base pairs would favour head-to-tail structures, whereas formation of psC:psC base pairs would be associated with a head-to-head strands orientation. Moreover, these substitutions may allow us to evaluate the effect of psC on the interactions between the C:C^+^ stack and G:T:G:T minor groove tetrads relative to the stacking preferences observed for the non-modified sequences.

**Figure 2:**
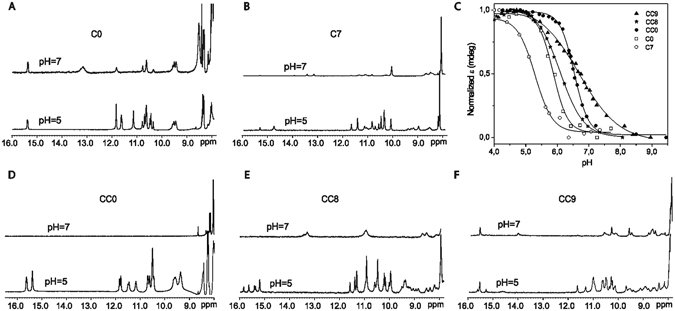
**CC9** forms i-motif structure at neutral pH. 1D ^1^H-NMR spectra at different pH. H_2_O/D_2_O 90:10, 25 mM phosphate buffer, 100 mM NaCl, T = 5 °C, [oligonucleotide] = 0.7–1 mM (**A**,**B**,**D**–**F**). CD pH titration of the 11-mer sequences were followed at the ellipticity maximum at pH 4: λ = 265 nm (**C0**, **CC8** and **CC9**) and λ = 265 nm (**C7** and **CC0**), 25 mM AcONa buffer, 100 mM NaCl, T = 5 °C, [oligonucleotide] = 20 μM (**C**).

**Figure 3:**
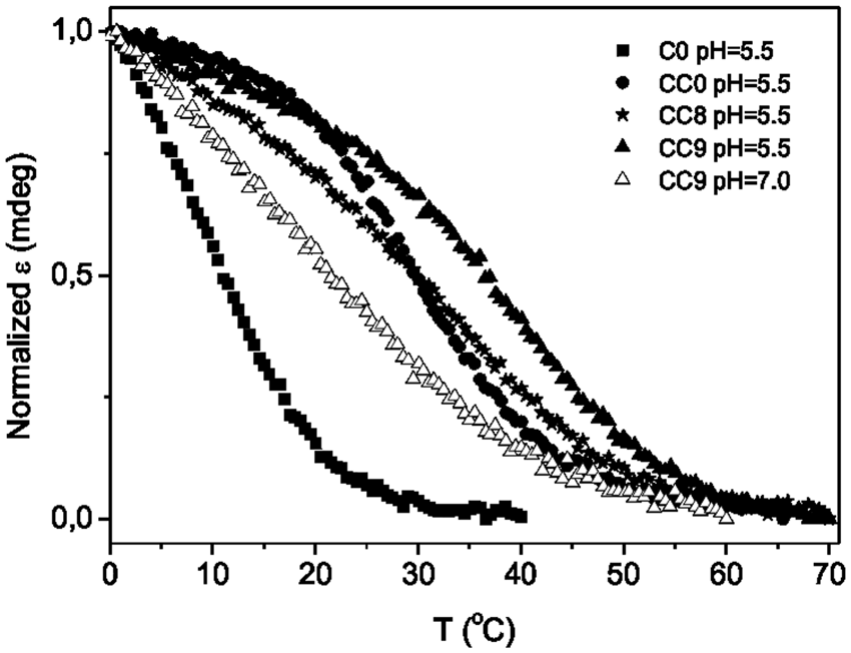
Thermal stability of psC-containing i-motifs. CD melting curves of **C0**, **C7**, **CC0**, **CC8** and **CC9**. Experimental conditions: [oligonucleotide] = 20 μM, 25 mM of buffer solution (cacodylate (pH 5.5) or phosphate (pH 7) and 100 mM NaCl.

**Figure 4:**
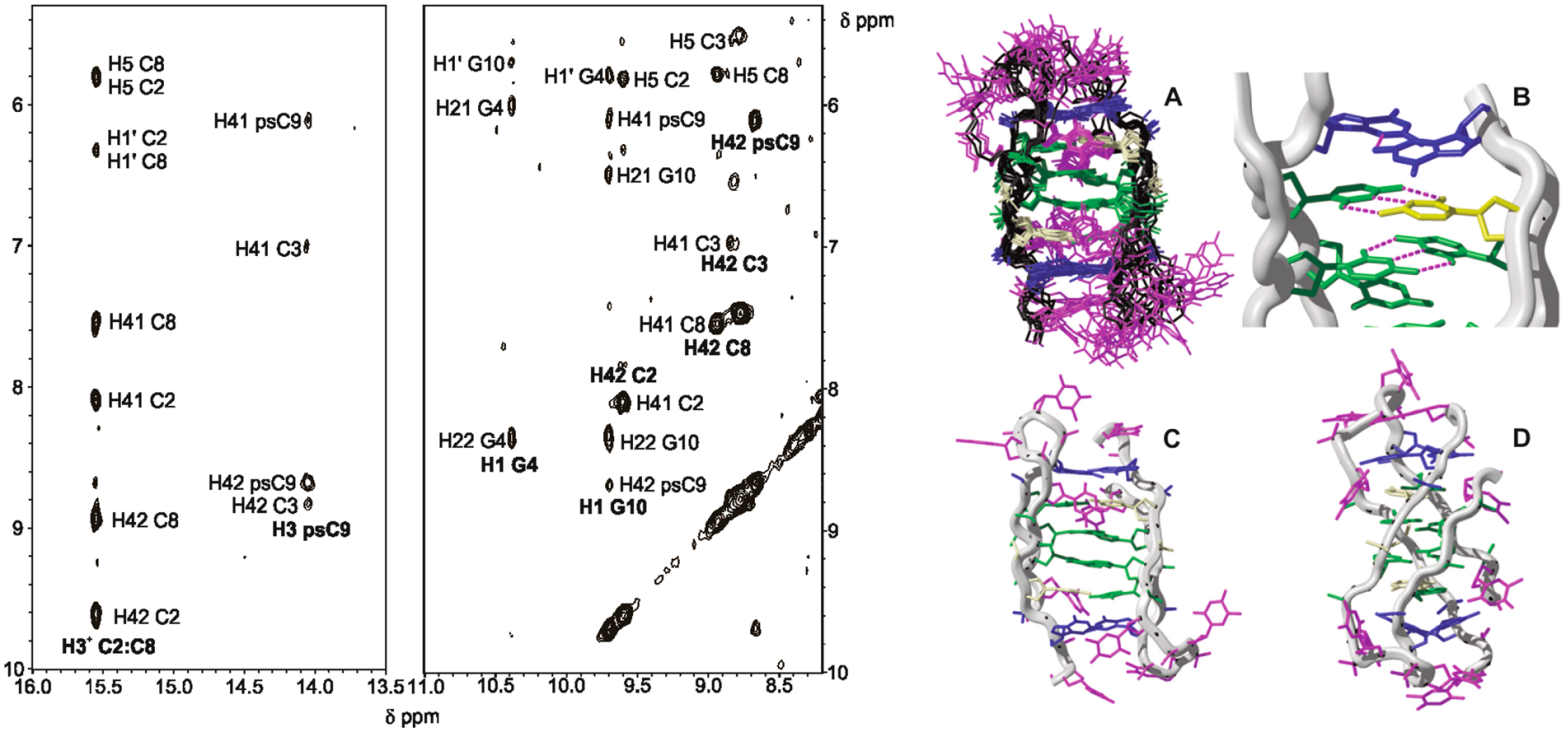
Assignment of exchangeable protons and solution dimeric structure of **CC9**. Left: Imino and amino exchangeable protons region of NOESY spectrum (250 ms) of **CC9**. H_2_O/D_2_O 90:10, 25 mM phosphate buffer pH 7, 100 mM NaCl, T = 5 °C, [oligonucleotide] = 0.8 mM. Right: (**A**) Structural ensemble. (**B**) Detail of C:C^+^, C:psC and G:G stack. (**C**,**D)** Front and lateral view of a representative structure. Cytosines are shown in green, psC in yellow, guanines in blue and thymines in red.

## Results and Discussion

### psC-containing sequences form i-motif structures

For the three psC-containing sequences, 1D NMR spectra show the characteristic i-motif signals at around 15 ppm (see Fig. 2B,E and F and Supplementary Fig. S6). Whereas **CC9** forms an i-motif structure at neutral pH, **C7** and **CC8** require acidic conditions for i-motif formation. The pH behaviour of the non-modified sequences is also shown in Fig. 2(A and D) for comparison. In the case of the **C7**, incorporation of psC destabilizes the i-motif. At neutral pH, several imino proton signals between 13–13.5 ppm are observed, and the signals corresponding to C:C^+^ formation (~15 ppm) are only residual. Most probably, the signals at 13–13.5 are due to the formation of alternative stem-loop hairpin or duplex structures with Watson-Crick base-pairs. At lower pH, these signals disappear and those corresponding to C:C^+^ base pairs become more intense. For the 11-mers, a sequence-dependent effect is found. In the case of **CC9**, i-motif formation is favoured at neutral pH in comparison to the non-modified sequence, which does not exhibit the characteristic i-motif signals under these conditions. This stabilization effect is not observed for **CC8**, in which only broad signals around 13 and 11 ppm are observed at neutral pH.

Estimation of the effective pH_T_ values (midpoint of the pH transition) was carried out by pH titration monitored by circular dichroism (see Table 1 and Fig. 2C). At 20 μM oligonucleotide concentration, the lower values were obtained for **C0** (5.84) and **C7** (5.33). In the case of the 11-mer sequences, pH titration of **CC8** afforded the lower value (6.1), whereas pH_T_ of **CC9** (6.7) was slightly higher than that obtained for the unmodified sequence **CC0** (6.5). Moreover, **CC9** exhibits a much broader pH transition range than **CC0** and **CC8**. The ample pH transitional range and the increased stability of CC9 make this i-motif structure stable at neutral pH. Consequently, we focused in study its structure in detail by 2D NMR methods.

**Table 1:** Melting temperature and pH_T_ values of **C0**, **C7**, **CC0**, **CC8** and **CC9**.

Name	Sequence	pH_T_	T_m_ (°C) pH 5.5	T_m_ (°C) pH 7
C0	d(TCGTTTCGT)	5.8 ± 0.1	~10	—
C7	d(TCGTTTpsCGT)	5.3 ± 0.1	—	—
CC0	d(TCCGTTTCCGT)	6.5 ± 0.1	29.6 ± 0.1	—
CC8	d(TCCGTTTpsCCGT)	6.1 ± 0.1	28.5 ± 0.1	—
CC9	d(TCCGTTTCpsCGT)	6.7 ± 0.1	37.4 ± 0.1	16.7 ± 0.1

Experimental conditions: [oligonucleotide] = 20 μM, 25 mM of buffer solution (cacodylate (pH 5.5) or phosphate (pH 7)) and 100 mM NaCl.

The thermal stability of the modified oligonucleotides, and their controls was studied by CD and NMR melting experiments (see Table 1 and Figs 3 and S7). In most cases, the thermal stability of the modified i-motifs is lower than the unmodified ones. Only in the case of **CC9**, a significant stabilization is observed, being this effect more pronounced at neutral pH. As expected from pH_T_ values, C0 and C7 at low oligonucleotide concentration are basically unstructured even at pH 5.5.

The stabilization observed for CC9 is not as high as that observed for 2′-F-araC substitutions^25^, but comparable with the effect of 5-methylcytosines^22^, LNA^23^ and other stabilizing sugar substitutions^31^. As in the case of LNA^32^, the effect of psC substitutions on the overall pH_T_ is highly dependent on their particular position. The same occurs with the thermal stability. Whereas psC located at the ends of the C-stack stabilizes the i-motif, substitutions in central position provoke the opposite effect. This destabilization is similar to that found for 5-hydroxymehtylcystosine^22^ or UNA^33^, and not as pronounced as other destabilizing modifications like acyclic threoninol derivatives^34^.

### CC9 i-motif structure at neutral pH is a head-to-tail dimer

In the case of **CC9**, pseudoisocytidine exchangeable proton signals could be identified by imino-amino NOE connections lacking H41/H42-H5 cross-peaks. NOESY spectra confirm the formation of the C3:psC9 base-pair, as well as C:C^+^ base-pairs between non-equivalent cytosines which were assigned to C2 and C8. This assignment is only consistent with a head-to-tail orientation of the two subunits in the dimer (see Supplementary Fig. S8). The stacking order T11-G10-psC9-C2-C8-C3-G4-T5 (3′-E topology) can be verified by a number of cross-peaks involving exchangeable protons (H3^+^C2:C8-H3psC9, H41/H42psC9-H1G10, H41/H42C3-H1G4, H42-H2′/H2″C8 and MeT5/T11-H1′G4/G10) (see Fig. 4 and Supplementary Fig. S9). The presence of G:G base pair is supported by H1G4-H1′G10 and H1G10-H1′G4 NOEs. Other cross-peaks clearly indicate that G:G base pairs are stacked on top of the C:psC pairs. In contrast to the unmodified sequence, no cross-peaks corresponding to the formation of G:T base pairs were observed at any pH value.

On the basis of NOESY cross-peaks, the solution structure of **CC9** at neutral pH was generated by restrained molecular dynamics calculations on the basis of 128 distance constraints. The resulting structural ensemble is shown in Fig. 4, Right (A) (PDB code: 5NIP). With the exception of the thymine residues, the structure is well-defined and consists of two DNA loops that self-associate through two central C:C^+^ base pairs, flanked by two psC:C base pairs and capped by two G:G base pairs. Interestingly, no G:T:G:T tetrad is formed, indicating that the stabilizing effect due to the incorporation of neutral psC:C as capping base pairs of the C:C^+^ stack compensates the minor groove tetrad breakdown.

Additional three small signals at around 15 ppm were detected in the exchangeable proton spectra of CC9 at low pH, indicating the presence of i-motif minor species (see Fig. 2F). Only two cross-peaks between each of these imino signals and cytosine amino protons are observed, indicating the formation of C:C^+^ base pairs between equivalent residues. Amino protons of two of these cytosines, assigned to C2 and C8, show cross-peaks with their own H2′/H2″ protons, indicating that these residues are located in 3′-3′ stacked C:C^+^ base pairs. Overlapping with the major species signals did not allow to complete the assignment of the minor species. However, the observed cross-peaks are consistent with a head-to-head dimer with the same stacking order.

### Equilibria between head-to-tail and head-to-head i-motif dimers are found for C7 and CC8

In contrast to the well-defined structure of **CC9**, **CC8** exhibits a very different behaviour. **CC8** sequence was designed for psC-containing base pairs to occupy central positions in the i-motif. However, such structure is not found at any pH. Whereas no i-motif is observed at neutral conditions, a complex equilibrium between different i-motif species is found below pH 6 (up to eight cytosine and two pseudoisocytidine residues involved in structured species are found). Under these conditions, NMR spectra are consistent with the formation of C:psC^+^ and psC:psC^+^ base pairs (see Supplementary Fig. S10), instead of neutral C:psC base pairs. Interestingly, signals corresponding to G:T:G:T minor groove tetrads are observed (see Supplementary Fig. S10). The multiple structures formed may include dimers with the same strand orientation but different stacking order. The major species could be assigned to a head-to-head dimeric structure, containing three C:C^+^ and one psC:psC^+^ hemiprotonated base pairs with the stacking order T11-G10-C9-C2-psC8-C3-G4-T5 (see Supplementary Fig. S11). Imino proton signals corresponding to psC residues are significantly downshifted. These results confirm a preference for maximizing the number of C:C^+^ base pairs, when neutral psC:C base pairs cannot occupy the external positions in the intercalation stack. Head-to-tail structures are not favoured even at acidic pH. Since such structure requires the formation of two C:C^+^ and two psC:C^+^ base pairs, the preference for the head-to-head conformer (with three C:C^+^ and one psC:psC^+^ base pairs) is most probably a consequence of the lower p*K*_a_ of pseudoisocytidine.

In the case of **C7** at pH 5, at least four different species coexist in slow equilibrium, according to the number of cytosine H5-H6 cross-peaks of the TOCSY spectrum. Based on exchangeable protons cross-peaks (see Supplementary Figs S12 and S13), the most intense set of signals could be assigned to two different i-motif species: head-to-tail (containing C:psC^+^ base pairs, imino signal at 14.70 ppm) and head-to-head (containing one C:C^+^ base pair, imino signal at 15.27 ppm, and presumably, one psC:psC^+^ base pair). Both species exhibit the same stacking order (Supplementary Fig. S14) and are capped by G:T:G:T minor groove tetrads. The signal corresponding to the psC:psC^+^ base pair (15.66 ppm) is only observable at pH 4, under which conditions the head-to-head structure is predominant (see Supplementary Fig. S13). The fact that the head-to-tail structure is not observed at neutral pH, reveals that the i-motif cannot be formed only by neutral psC:C base pairs. Some protonated base pairs are required for the stabilization of the i-motif structure.

### The effect of pseudoisocytidine incorporation in other i-motif forming sequences

**Figure 5:**
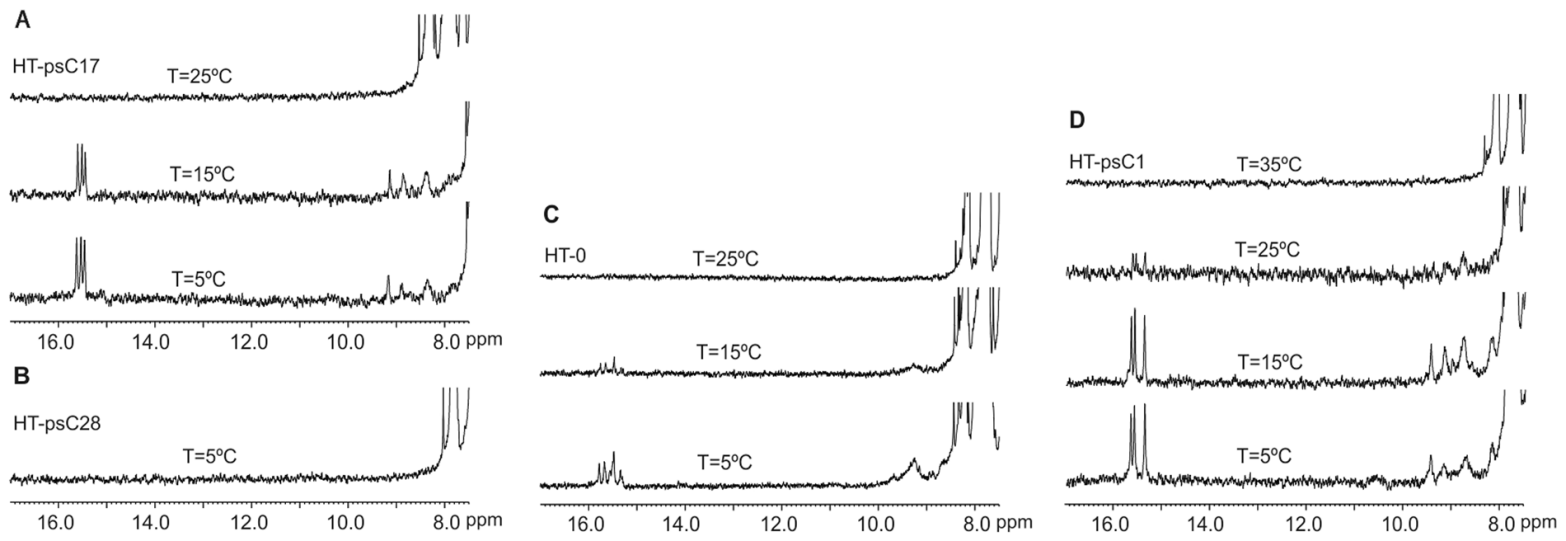
Single psC residue at position 1 in HT sequence stabilizes i-motif structure at neutral pH. Series of NMR spectra at different temperature of: (**A**) **HT-psC17**, (**B**) **HT-psC28**, (**C**) **HT-0** and (**D**) **HT-psC1**. [oligonucleotide] = 100 μM, 10 mM phosphate buffer, pH 7.

To evaluate the generality of our results, we explored the effect of site-specific replacements of cytidine by psC residues in the human telomeric sequence (CCCTAA)_3_CCC. Three modified sequences **HT-psC1**, **HT-psC17** and **HT-psC28**, incorporating a) a single pseudoisocytidine residues at position 1 (**HT-psC1**), b) two double substitutions at position 1 and 7 (**HT-psC17**) and 2, and 8 (**HT-psC28**) were studied by ^1^H-NMR and CD melting curves. According to the three-dimensional structure of the native telomeric sequence^7^, psC residues are in a central position in the C:C^+^ stack in **HT-psC28**, and in external positions in **HT-psC1** and **HT-psC17**. CD and NMR melting experiments at acidic pH indicate that all substitutions are destabilizing, although the single substitution in the 5′-terminal cytosine has a very small effect (Figs S15 and S16). The stability of this i-motif at neutral pH is small and, consequently, melting T_1/2_ values are low (14.0, 14.2 and 11.3 °C ( ± 0.1 °C) for **HT-0**, **HT-psC1** and **HTpsC2**, respectively) and contain considerable errors. However, NMR results indicate that incorporation of psC at C1 position clearly confers a higher stability to the i-motif structure (Fig. 5). The effect is also visible for **HT-psC17**, although to a lesser extension. In these conditions, the double psC substitution in central position destabilizes the i-motif completely.

## Conclusions

We have shown that the effect of replacing cytidine residues by psC strongly depends on the location of the cytidine residues. Formation of neutral psC:C base pairs has a stabilizing effect when located at the end of the C:C^+^ stacks, but destabilizes the i-motif in central positions. I-motifs containing psC residues in central positions are only found at low pH, and require the formation of psC:C^+^ and psC:psC^+^ hemiprotonated base pairs. These i-motifs are less stable than the unmodified sequences. These results indicate that the presence of positive charges in the i-motif core is essential to stabilize this non-canonical structure. Interestingly, formation of G:T:G:T minor groove tetrads in an i-motif context seems to require the presence of a protonated base pair nearby, suggesting that the stabilization provoked by these tetrads may be due to cation-pi interactions.

## Methods

### Oligonucleotides synthesis

Oligodeoxynucleotides were synthesized on an ABI 3400 DNA synthesizer by using standard solid-phase phosphoramidite chemistry at 1 μmol scale. Some minor modifications were required in the synthesis cycle for the psC-containing sequences. Rather than 1*H*-tetrazole, a stronger activator agent, 5-Benzylthio-1*H*-tetrazole (BTT) was used, together with an extended coupling time of 15 min for the incorporation of psC residues. As oxidizing agent, 1 M solution of ^t^BuOOH in DCM was used. Cleavage from the solid support and nucleobases deprotection were carried out with concentrated aqueous ammonium hydroxide at room temperature for 12 or 24 h. Crude products were purified by ion exchange HPLC (250 × 4 mm NucleoPac PA-100 column from Dionex, solvent A: 1 M AcONH_4_ pH = 7 containing 7.5% ACN, solvent B: 7.5% ACN solution in H_2_O). Oligonucleotides were further desalted by EtOH precipitation and characterized by MS-MALDI-TOF. Synthesis results are summarized in Supplementary Table S1.

### Mass spectrometry

MS-MALDI-TOF spectra were acquired in the negative ion mode on an ABSciex 4800 plus device (see Supplementary Figs S17–S24). Samples were prepared by mixing 1μL of oligonucleotide solution (100–500 μM) with 1μL of ammonium citrate (50 mg/mL) and allowed to interact for few seconds. Next 1 μL of the mixture and 1μL of the matrix (2,4,6-trihidroxyacetophenone, THAP, 10 mg/mL in H2O/ACN 1:1) were mixed and deposited onto the plate.

### CD and UV spectroscopy

Circular dichroism spectra were recorded on a Jasco J-810 spectropolarimeter fitted with a thermostated cell holder. CD spectra were recorded in 25 mM sodium phosphate buffer or 25 mM sodium acetate buffer at different pH values and in presence of 100 mM NaCl. Samples were initially heated at 90 °C for 5 min, and slowly allowed to cool to room temperature an stored at 4 °C until use. For pH titration experiments, the pH was adjusted by adding aliquots of concentrated solutions of HCl or NaOH.Molar extinction coefficients for the psC-containing sequences (see Supplementary Table S1) were calculated by applying the nearest-neighbour method and considering the same molar extinction coefficient for cytidine and pseudoisocytidine residues.

### NMR

Samples for NMR experiments were dissolved (in Na^+^ form) in either D_2_O or 9:1 H_2_O/D_2_O (25 mM sodium phosphate buffer) in presence of 100 mM NaCl. Experiments were carried out at different pH values, ranging from 3.5 to 7. The pH was adjusted by adding aliquots of concentrated solution of either DCl or NaOD. All NMR spectra were acquired in Bruker spectrometers operating at 600 and 800 MHz, equipped with cryoprobes and processed with the TOPSPIN software. In the experiments in D_2_O, presaturation was used to suppress the residual H_2_O signal. A jump-and-return pulse sequence^35^ was employed to observe the rapidly exchanging protons in 1D H_2_O experiments. NOESY^36^ spectra in D_2_O and 9:1 H_2_O/D_2_O were acquired with mixing times of 150, 250 and 300 ms. TOCSY^37^ spectra were recorded with the standard MLEV-17 spin-lock sequence and a mixing time of 80 ms. In most of the experiments in H_2_O, water suppression was achieved by including a WATERGATE^38^ module in the pulse sequence prior to acquisition. The spectral analysis program SPARKY was used for semiautomatic assignment of the NOESY cross-peaks and quantitative evaluation of the NOE intensities.

### Assignment of the NMR spectra

A complete 2D NMR study at different pH of all the sequences was carried out to characterize the different i-motif structures formed in each case. The NMR assignment of the unmodified sequence **C0** has been previously reported^15^. The NMR spectra of **CC0** exhibit very similar features, and the assignment of the two co-existing species (head-total and head-to-head) could be carried out as described in the supplementary material. Assignment of the NMR spectra of the modified 11-mers, **CC8** and **CC9**, was carried out in a similar way. The NMR spectrum of **CC9** was assigned at neutral pH. In addition to the i-motif signals, some extra signals were observed, probably corresponding to unfolded or partially folded species. For this second species (see Supplementary Fig. S9), C2, C8, C3 and psC9 residues could be identified. However, cross-peaks involving exchangeable protons were only found for C3 and psC9. The relative intensity of this minor species decreases at higher oligonucleotide concentration, indicating that this species is monomeric.

### NMR constraints

Qualitative distance constraints were obtained from NOE intensities. NOEs were classified as strong, medium or weak, and distances constraints were set accordingly to 3, 4 or 5 Å. In addition to these experimentally derived constraints, hydrogen bond and planarity constrains for the base pairs were used. Target values for distances and angles related to hydrogen bonds were set to values obtained from crystallographic data in related structures^39^. Due to the relatively broad line-widths of the sugar proton signals, J-coupling constants were not accurately measured, but only, roughly estimated from DQF-COSY cross-peaks. Loose values were set for the sugar dihedral angles δ, ν_1_ and ν_2_ to constrain deoxyribose conformation to North or South domain.

### Structural calculations

Structures were calculated with the program DYANA^40^ and further refined with the SANDER module of the molecular dynamics package AMBER 12.0. Resulting DYANA structures were taken as starting points for the AMBER refinement, consisting of an annealing protocol in vacuo, followed by trajectories of 500 ps each in which explicit solvent molecules were included and using the Particle Mesh Ewald method to evaluate long-range electrostatic interactions. The specific protocols for these calculations have been describe elsewhere^41^. The BSC1 force field^42^ was used to describe the DNA, and the TIP3P model was used to simulate water molecules. Charges for pseudoisocytosine were calculated using ab initio calculations with RESP/6-31G(d) in single base models. Analysis of the representative structures was carried out with the program MOLMOL^43^. The refined structures are deposited in the PDB (code: 5NIP).

### Data availability

All data generated or analysed during this study are included in this published article (and its Supplementary Information files).

## Electronic supplementary material

Supplementary PDF File
