# Supplementary material for: The effect of the neutral cytidine protonated analogue pseudoisocytidine on the stability of i-motif structures
Source: Sci Rep. 2017 Jun 5;7:2772. doi: 10.1038/s41598-017-02723-y (PMC5459817; doi:10.1038/s41598-017-02723-y)
Supplement: Supplementary file 1 — Supplementary PDF File [file 41598_2017_2723_MOESM1_ESM.pdf]

ELECTRONIC SUPPLEMENTARY INFORMATION

The effect of the neutral cytidine protonated analogue pseudoisocyitidine on the stability of i-motif structures

B. Mir,[a] X. Solés,[a] C. González[b,c] and N. Escaja[a,c]

[a] *Inorganic and Organic Chemistry Department, Organic Chemistry Section, and IBUB, University of Barcelona, Martí i Franquès 1-11, 08028 Barcelona, Spain.*
[b] *Instituto de Química Física Rocasolano, CSIC, Serrano 119, 28006 Madrid, Spain.*
[c] *BIOESTRAN associated unit UB-CSIC.*

1. NMR assignment details 3

2. Supplementary Figures

Figure S1: Amino and non-exchangeable protons region of NOESY spectrum of **CC0** 4
Figure S2: Imino-amino/aromatic protons regions of NOESY spectrum of **CC0** 5
Figure S3: Imino-sugar protons region of NOESY spectrum of **CC0** 6
Figure S4: Schemes of the head-to-head and head-to-tail i-motif species of **CC0** 7
Figure S5: psC:psC, psC:psC$^+$ and C:psC$^+$ base pairs 7
Figure S6: Series of 1D ^{1}H-NMR spectra of **C7**, **CC8** and **CC9** at different pH and T 8
Figure S7: Series of CD spectra vs. T of **C0**, **C7**, **CC0**, **CC8** and **CC9** 9
Figure S8: Scheme of the head-to-tail species of **CC9** 10
Figure S9: Amino/aromatic protons regions of the NOESY spectrum of **CC9** 10
Figure S10: Exchangeable protons regions of **CC8** 11
Figure S11: Schemes of the head-to-head and head-to-tail i-motif species of **CC8** 12
Figure S12: Exchangeable protons regions of **C7** 12
Figure S13: Imino protons regions of **C7** at different pH 13
Figure S14: Schemes of the head-to-head and head-to-tail i-motif species of **C7** 14
Figure S15: CD Melting curves of **HT0**, **HT-psC1**, **HT-psC17** and **HT-psC28**. 14
Figure S16: 1D ^{1}H-NMR spectra vs. T of **HT0**, **HTpsC1**, **HTpsC17** and **HTpsC28** 15
Figure S17: MALDI-TOF spectrum of **CC0** 16
Figure S18: MALDI-TOF spectrum of **CC9** 16
Figure S19: MALDI-TOF spectrum of **CC8** 16
Figure S20: MALDI-TOF spectrum of **C7** 17
Figure S21: MALDI-TOF spectrum of **HT0** 17
Figure S22: MALDI-TOF spectrum of **HT-psC1** 17
Figure S23: MALDI-TOF spectrum of **HT-psC17** 18
Figure S24: MALDI-TOF spectrum of **HT-psC28** 18

3. Supplementary Tables

Table S1: Oligonucleotide synthesis results 19
Table S2: Chemical shifts of non-exchangeable protons of **CC0**, pH 4 19
Table S3: Chemical shifts of exchangeable protons of **CC0**, pH 4 19
Table S4: Chemical shifts of the head-to-tail species of **CC9**, pH 7 20
Table S5: Chemical shifts of the major head-to-head species of **CC8**, pH 5 20
Table S6: Chemical shifts of the head-to-tail species of **C7**, pH 5 20
Table S7: Chemical shifts of the head-to-head species of **C7**, pH 4 21
Table S8: Experimental constraints and calculation statistics of **CC9** 21

1. NMR assignment details.

NMR assignment of the unmodified sequence d(TCCGTTTCCGT), **CC0**, was carried out at pH 4. The exchangeable proton spectra indicate that at this pH and T = 5°C, **CC0** is highly structured. Characteristic imino signals of hemiprotonated C:C$^+$ base pairs are observed at 15-16 ppm, confirming the formation of i-motif structures. However, much overlapping is found in the non-exchangeable protons spectra. Two broad cytosine H5-H6 cross-peaks (~7.85 and 7.40 ppm) are found in the TOCSY spectra, whereas up to twelve aromatic spin systems can be identified in the NOESY spectra. Since only eleven aromatic spin systems are expected for a unique dimeric symmetrical structure, we conclude that there are more than one species present. Fortunately, the exchangeable proton region exhibits very little overlapping and the cross-peaks patterns of imino and amino signals provide key structural information.

As shown in **Figure S1**, five different cytosine H41-H42 cross-peaks are found. All these amino proton signals exhibit cross-peaks with the imino signals observed at 15.48, 15.25 and 15.23 ppm (see **Figure S2**). One of these amino cross-peaks (the most intense) could be assigned to the amino protons of C2 and C8, which present degenerated signals. These amino protons only show cross-peaks with the imino signal at 15.48 ppm. The other four H41-H42 cross-peaks were assigned to two different pairs of C3 and C9 cytosine residues, indicating the formation of two i-motif species. One pair of amino protons (assigned to C3) shows cross-peaks with the imino signal at 15.25 ppm, whereas the other three pairs of amino protons exhibits cross-peaks with the imino signal at 15.23 ppm. Observation of H42C2/C8-H2'/H2"C2/C8 cross-peaks, characteristic of 3'-3' stacked C:C$^+$ base pairs, confirms that these residues are located in central positions in the two species. On the other hand, the existence of four sets of H41/H42C3-H1G4 and H41/H42C9-H1G10 cross-peaks reveals that these cytosine residues are located at the end of the C:C$^+$ tract, and are stacked with contiguous guanine residues. The good dispersion of these cross-peaks, together with the observed amino-imino cross-peaks pattern, allowed the assignment of C3 and C9 residues to each of the observed species. We conclude that the two i-motif dimeric species maintain the same stacking order (see **Figure S4)**, but have different strand orientation: head-to-head and head-to-tail. For the head-to-head species, hemiprotonated C:C$^+$ base pairs are formed between equivalent cytosines and each imino proton show cross-peaks with an unique amino pair, 15.25(C3) and 15.23(C9) ppm. In contrast, in the head-to-tail orientation, these base pairs are formed between non-equivalent cytosines and each imino signal shows cross-peaks with two different pairs of amino protons, signal at 15.23 ppm (C3 and C9). In spite of the different orientation, the chemical environment of the central base pairs is very similar in both species. The degenerated signals observed for C2 and C8 is consequence of this similarity.

Imino-imino cross-peaks between guanine and thymine residues, characteristic of G:T base pairs formation, are observed in both species. Moreover, H1-H1' contacts between guanine residues (G4/10-G4/10, for the head-to-head, and G4/10-G10/4 for the head-to-tail species) are observed, indicating an interaction between guanine residues through their minor groove side, and supporting the formation of two G:T:G:T minor groove tetrads, each at one side of the i-motif.

Assignment of the NMR spectra of the modified 11-mers, **CC8** and **CC9**, was carried out in a similar way as the unmodified sequence **CC0**. The most prominent features in the NMR spectra are explained in the main text.

2. Supplementary Figures.

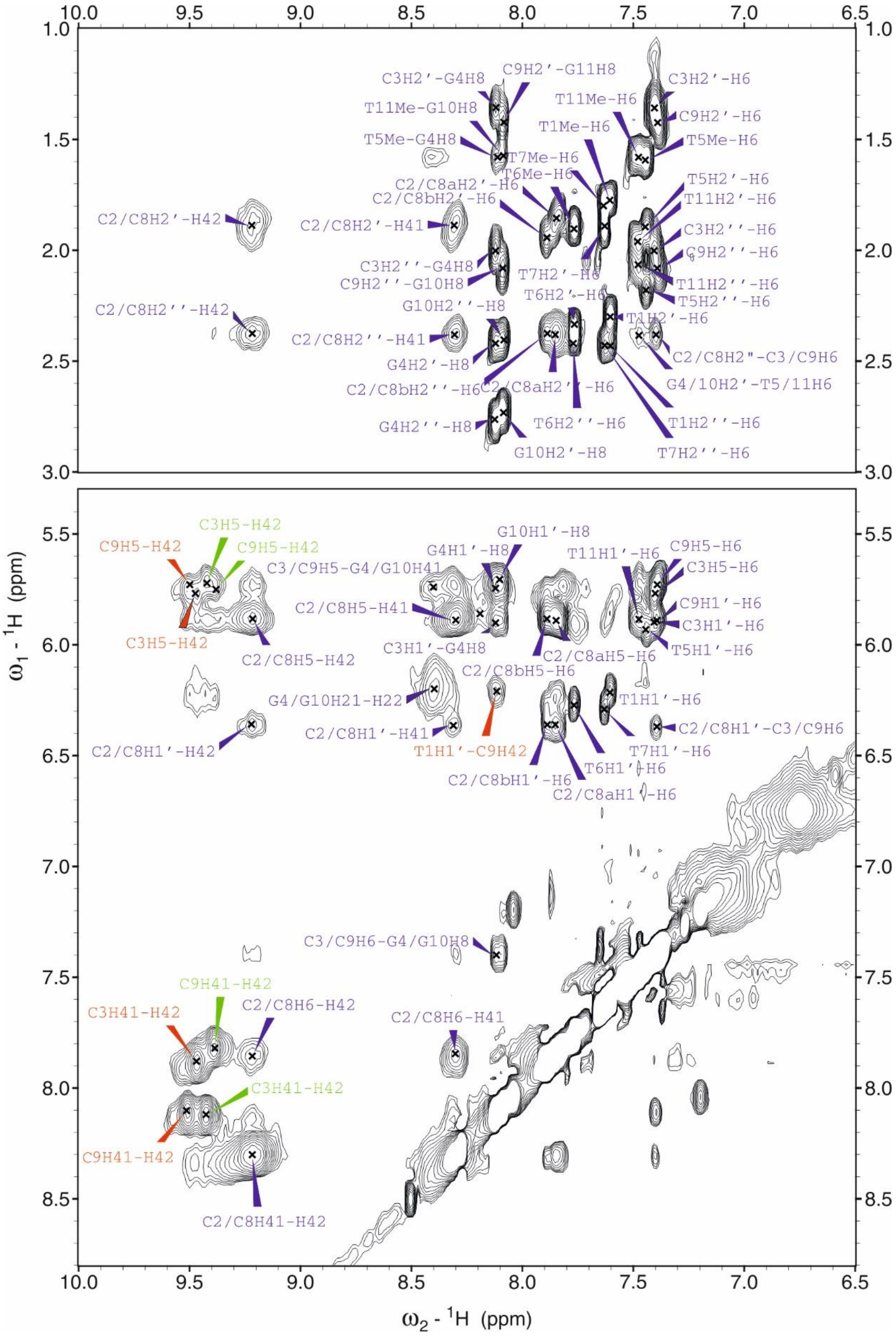

Figure S1. Amino and non-exchangeable protons region of NOESY spectrum (150 ms) of **CC0**. Ar-H1' and Ar-H2'/H2" region. H_2O/D_2O 90:10, 25 mM phosphate buffer pH 4, 100 mM NaCl, T=5°C, [oligonucleotide] = 0.8 mM. Red: head-to-head, green: head-to-tail, blue: both species.

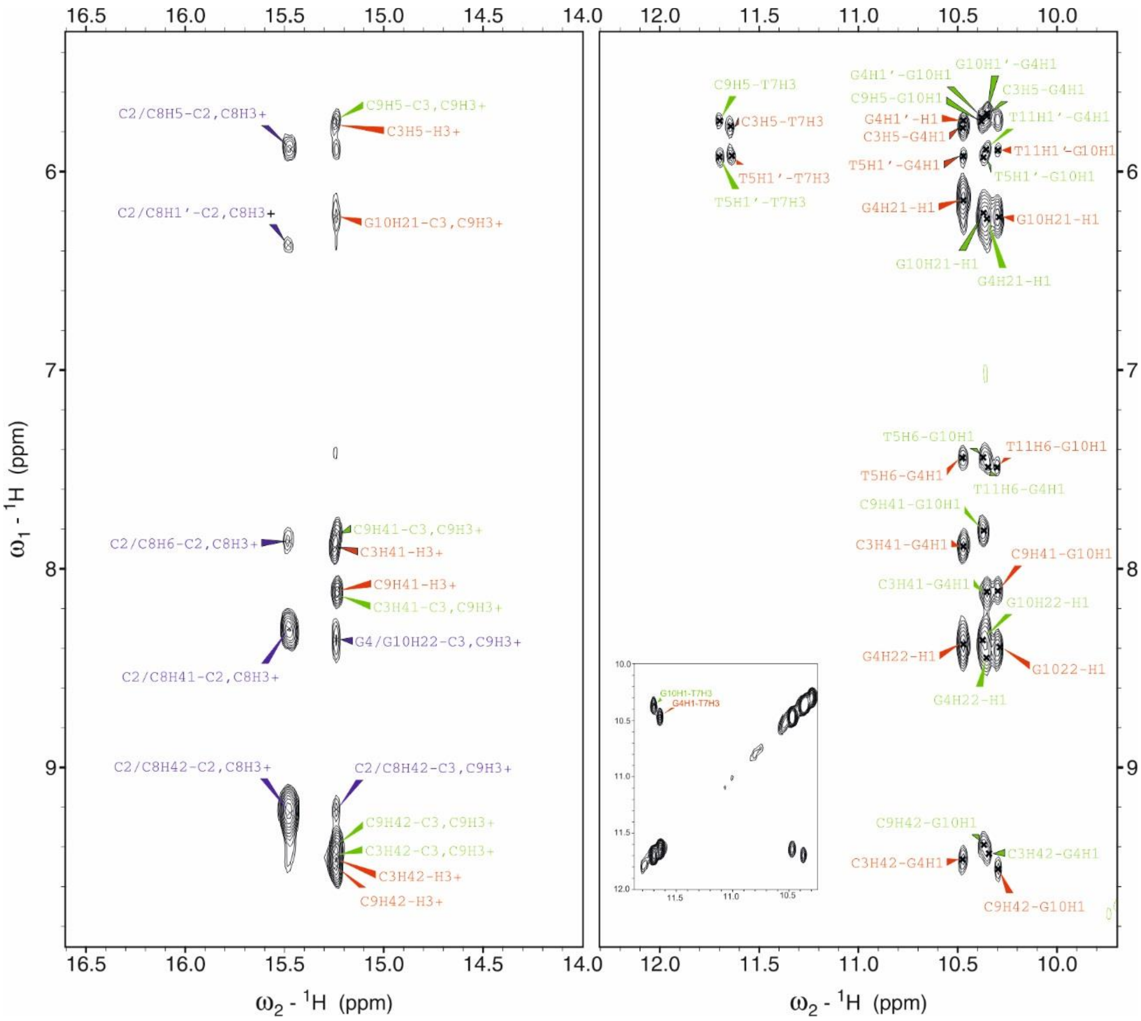

Figure S2. Exchangeable protons region of NOESY spectrum (150 ms) of **CC0**. H_2O/D_2O 90:10, 25 mM phosphate buffer, 100 mM NaCl, pH 4, T=5°C, [oligonucleotide] = 0.8 mM. Red: head-to-head species, green: head-to-tail species, blue: both species.

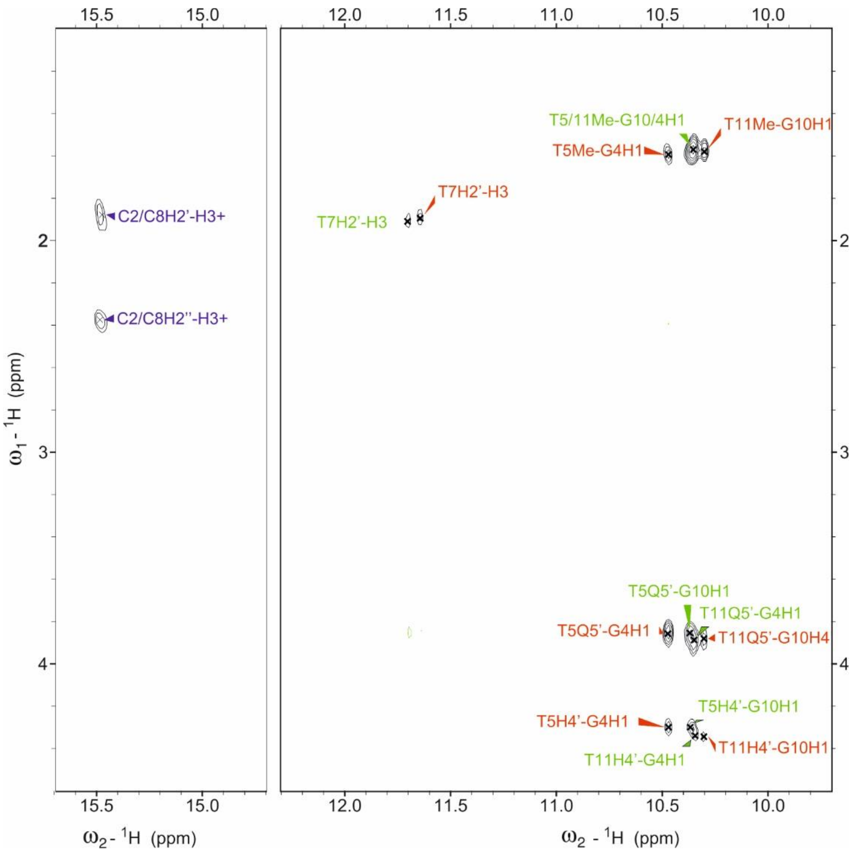

Figure S3. Exchangeable protons region (H3/H1-Me/H4'/H5'H5") of NOESY spectrum (150 ms) of **CC0**. H_2O/D_2O 90:10, 25 mM phosphate buffer pH 4, 100 mM NaCl, T=5°C, [oligonucleotide] = 0.8 mM. Red: head-to-head, green: head-to-tail, blue: both species.

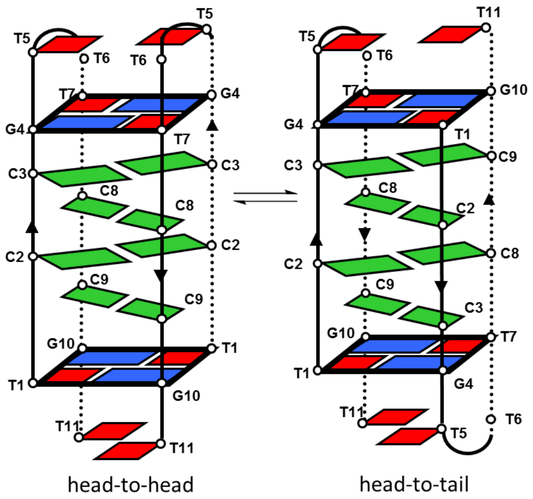

Figure S4.- Schematic models of the head-to head and head-to-tail dimeric i-motif structures of **CC0**.

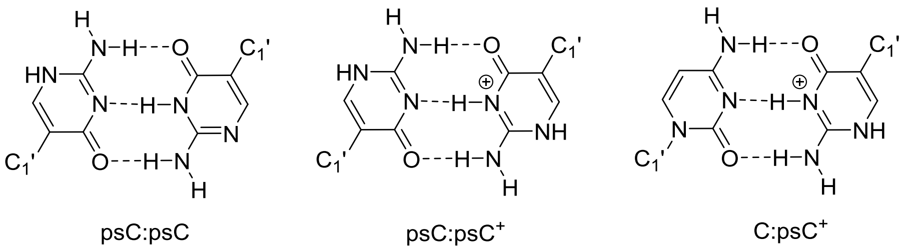

Figure S5. Neutral psC:psC base pair between the two tautomeric forms (left) and hemiprotonated psC:psC⁺ (center) and C:psC⁺ (right) base pairs.

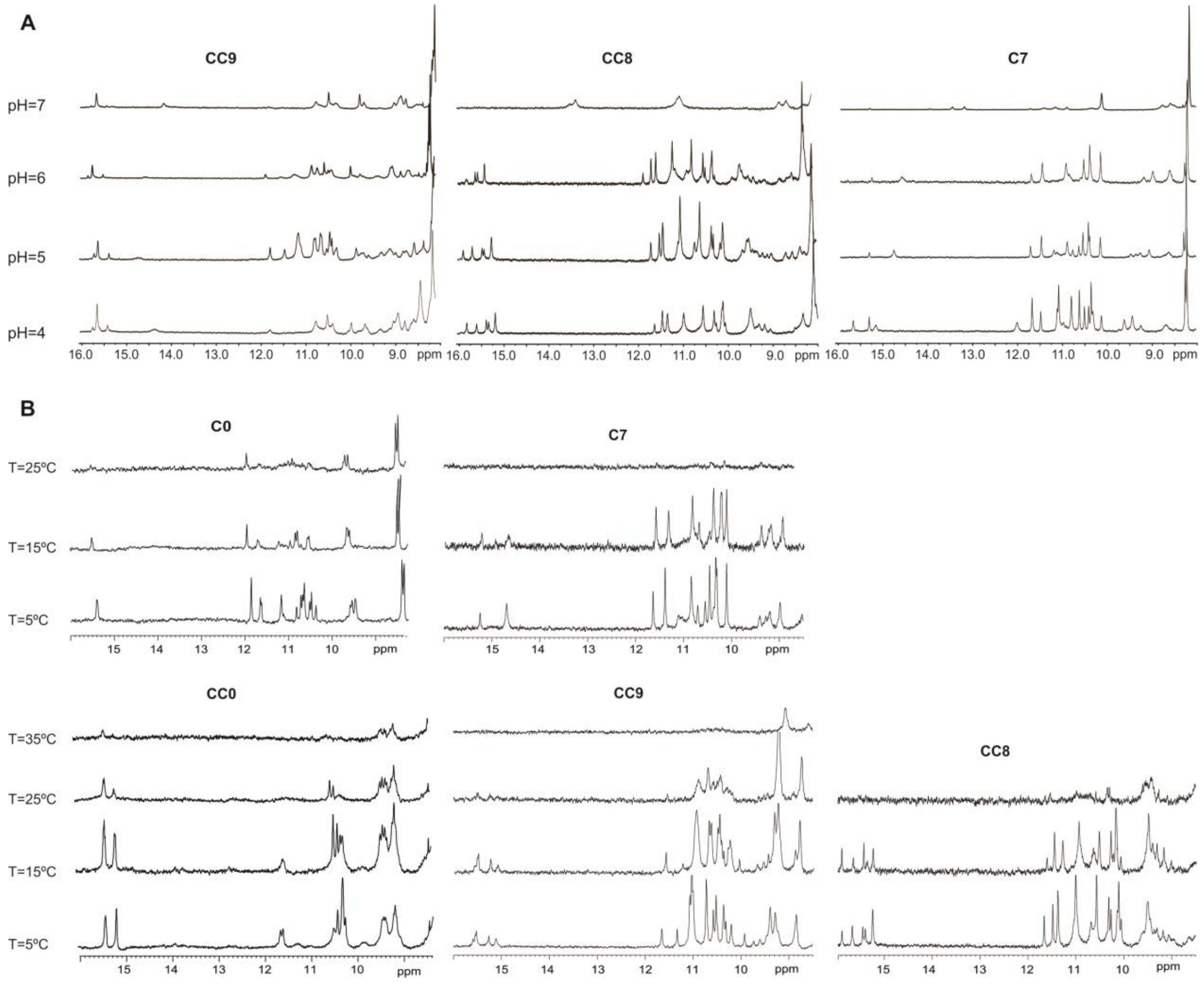

Figure S6. A: 1D ^{1}H-NMR spectra of **C7**, **CC8** and **CC9** at different pH and T=5°C. **B**: Series of NMR spectra of **C0**, **C7**, **CC0**, **CC8** and **CC9** at different temperature and pH 5.5. Experimental conditions: 25 mM phosphates, 100 mM NaCl, [oligonucleotide] = 0.8-0.95 mM.

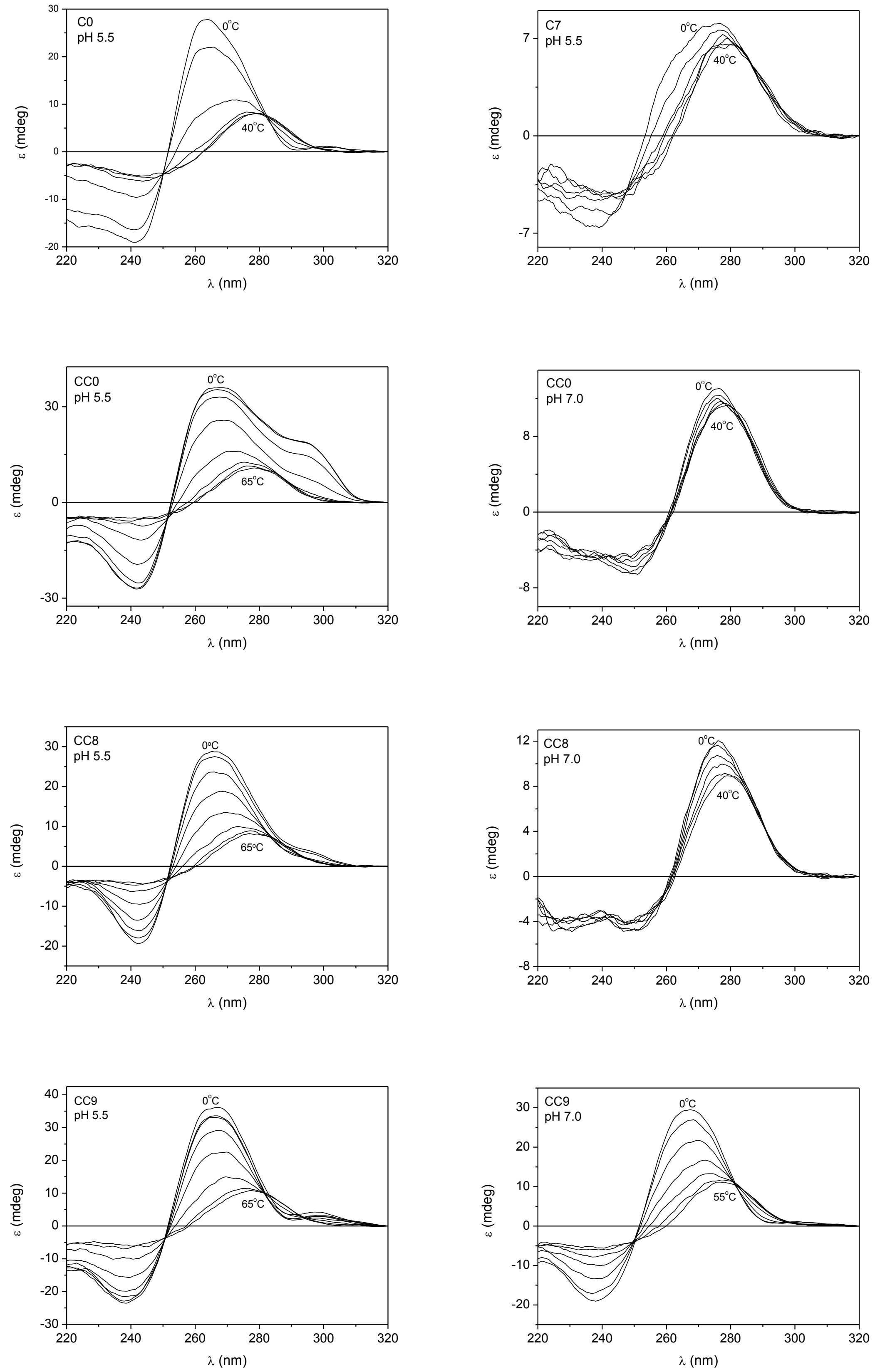

Figure S7. Series of CD spectra vs temperature of **C0**, **C7**, **CC0**, **CC8** and **CC9** at pH 5.5 and pH 7.0. Experimental conditions: [oligonucleotide] = 20 μM, 25 mM buffer (cacodylate pH 5.5 or phosphate pH 7.0).

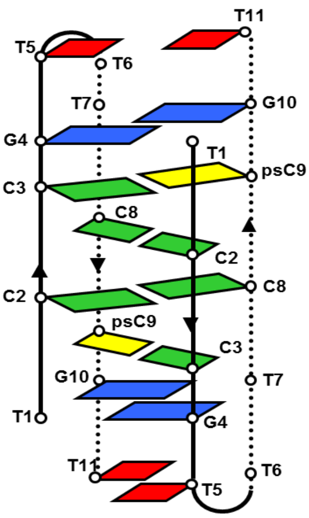

Figure S8. Scheme of the head-to-tail major i-motif species of **CC9** at neutral pH.

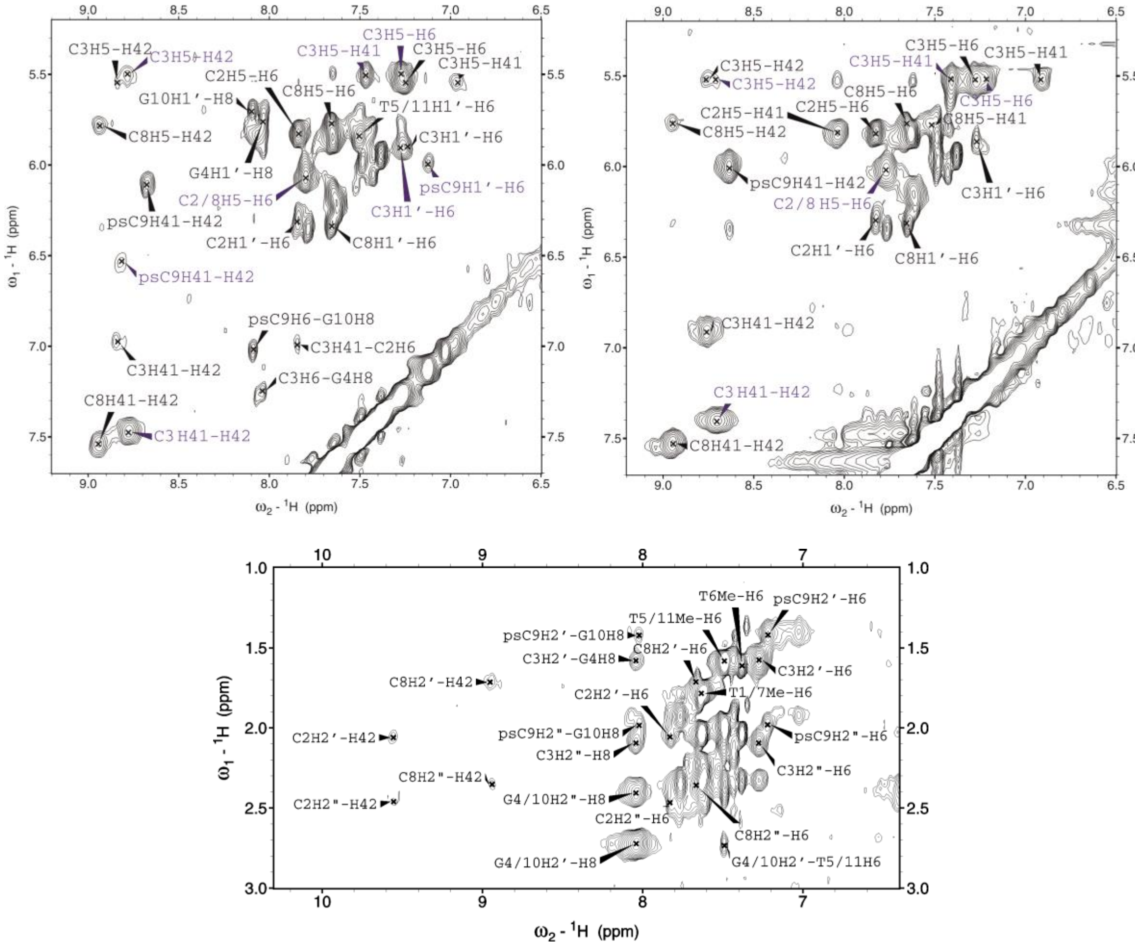

Figure S9. Regions of the NOESY spectra **of CC9** (H_2O/D_2O 90:10, phosphate buffer, 100 mM NaCl, T=5°C, pH 7). Top: At two oligonucleotide concentrations: 0.8 mM (left) and 1mM (right). Bottom: Aromatic-Me/H2'/H2" region, [CC9] = 1 mM. Black: head-to-tail i-motif species. Blue: Additional signals corresponding to partially structured monomeric species.

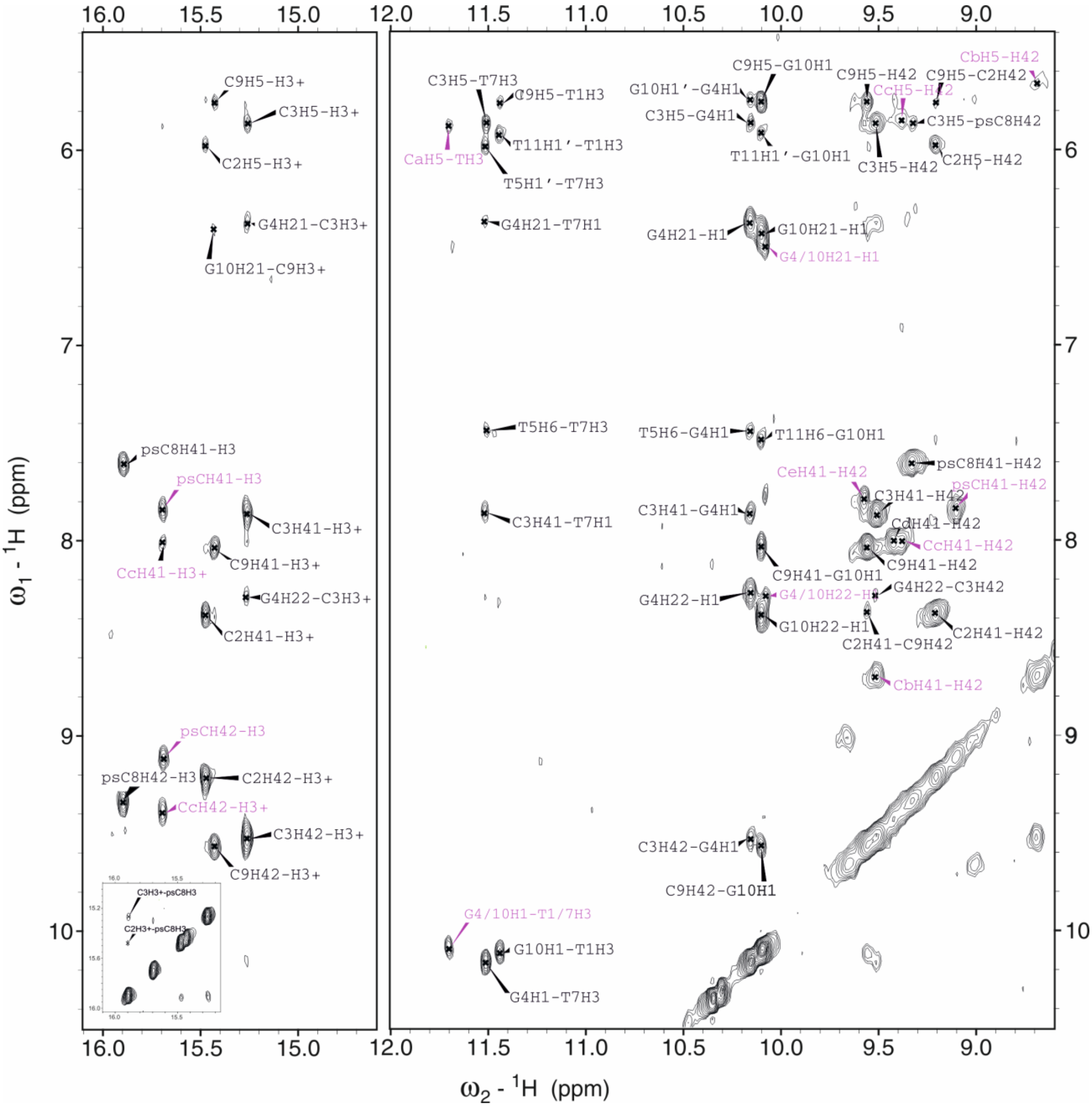

Figure S10. Exchangeable protons region of NOESY spectrum (150 ms) of **CC8**. H_2O/D_2O 90:10, 25 mM phosphate buffer pH 5, 100 mM NaCl, T=5°C, [oligonucleotide] = 0.8 mM. Black: head-to-head species with C9-C2-psC8-C3 stacking order. Magenta: additional signals corresponding to alternative i-motif species, head-to-head with different stacking order or head-to-tail.

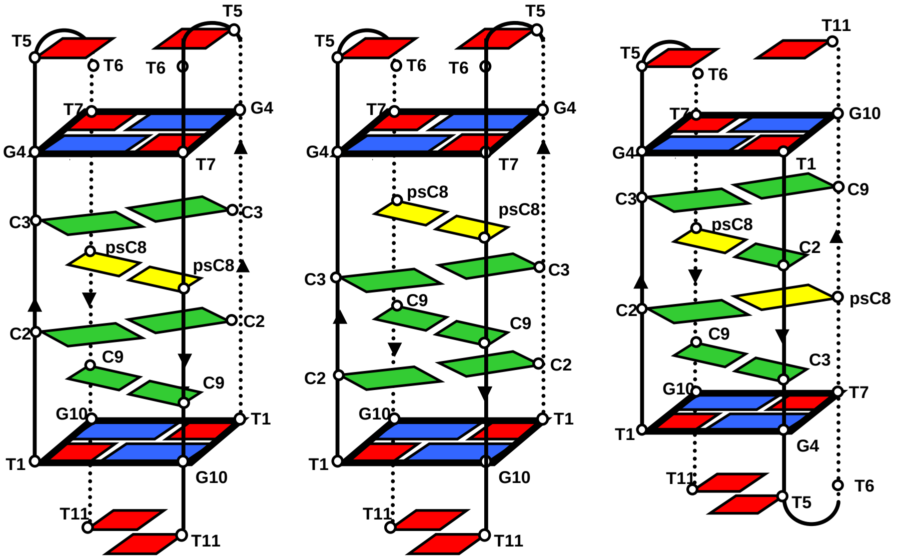

Figure S11. Schemes of the major head-to-head species of **CC8** (left) and the two other minor structures, compatible with observed NOESY cross-peaks: head-to-head with different stacking order (centre) and head-to-tail (right).

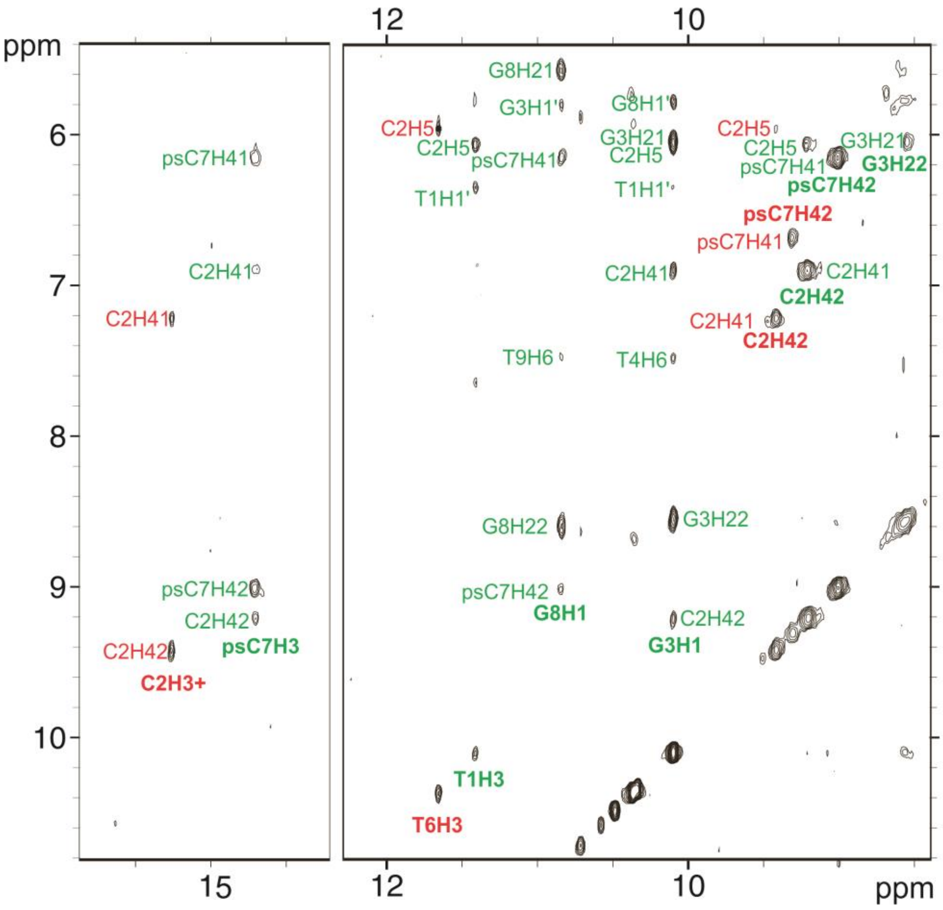

Figure S12. Exchangeable protons region of NOESY spectrum (150 ms) of **C7**. H_2O/D_2O 90:10, 25 mM phosphate buffer pH 5, 100 mM NaCl, T=5°C, [oligonucleotide] = 0.8 mM. Red: head-to-head species, green: head-to-tail species.

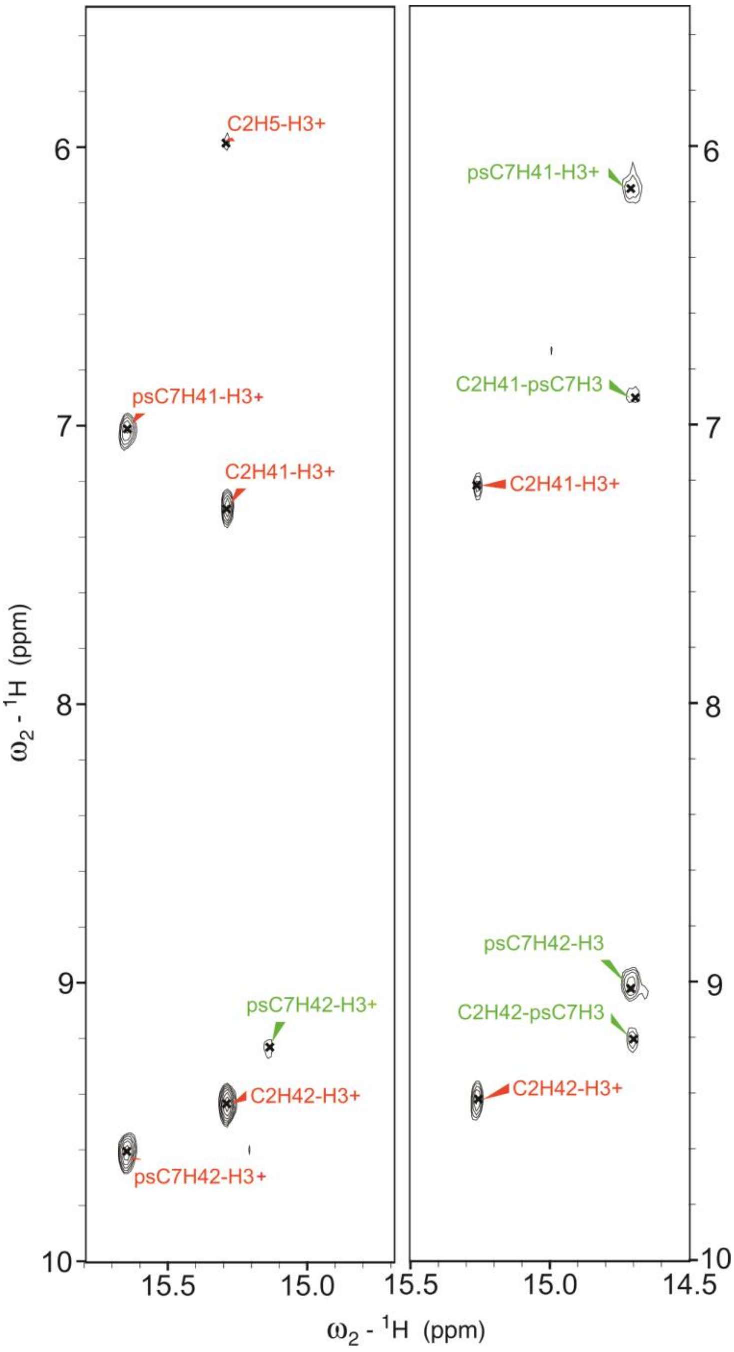

Figure S13. Imino protons region of NOESY spectrum (150 ms) of **C7**. H2O/D2O 90:10, 25 mM phosphate buffer, 100 mM NaCl, T=5°C, [oligonucleotide] = 0.8 mM at pH 4 (left) and pH 5 (right). Red: head-to-head species, green: head-to-tail species.

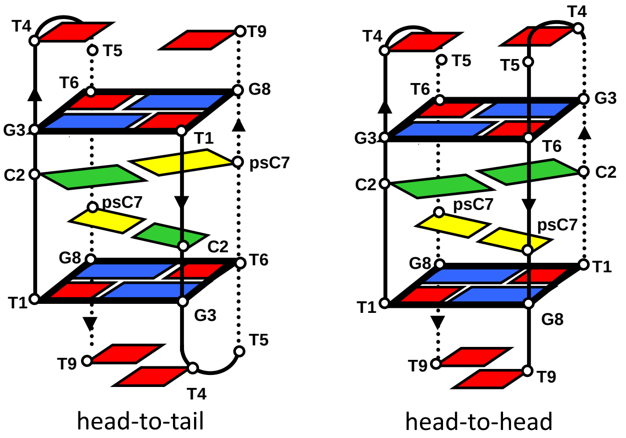

Figure S14. Schemes of the head-to-tail (left) and head-to-head (right) species of **C7**.

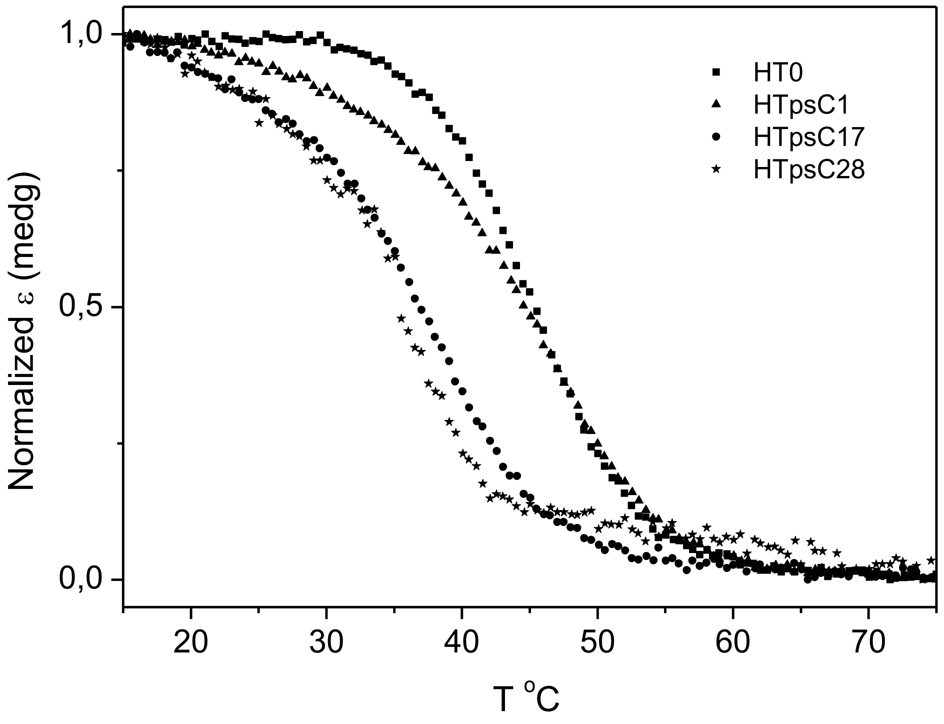

Figure S15. CD melting curves of the telomeric sequences at pH 5.5 at λ=287 nm. T_m experimental values: **HT0** (45.5°C), **HT-psC1** (43.0°C), **HT-psC17** (36.9°C) and **HT-psC28** (35.4°C). Experimental conditions: [oligonucleotide] = 20 μM, 25 mM cacodylate buffer pH 5.5.

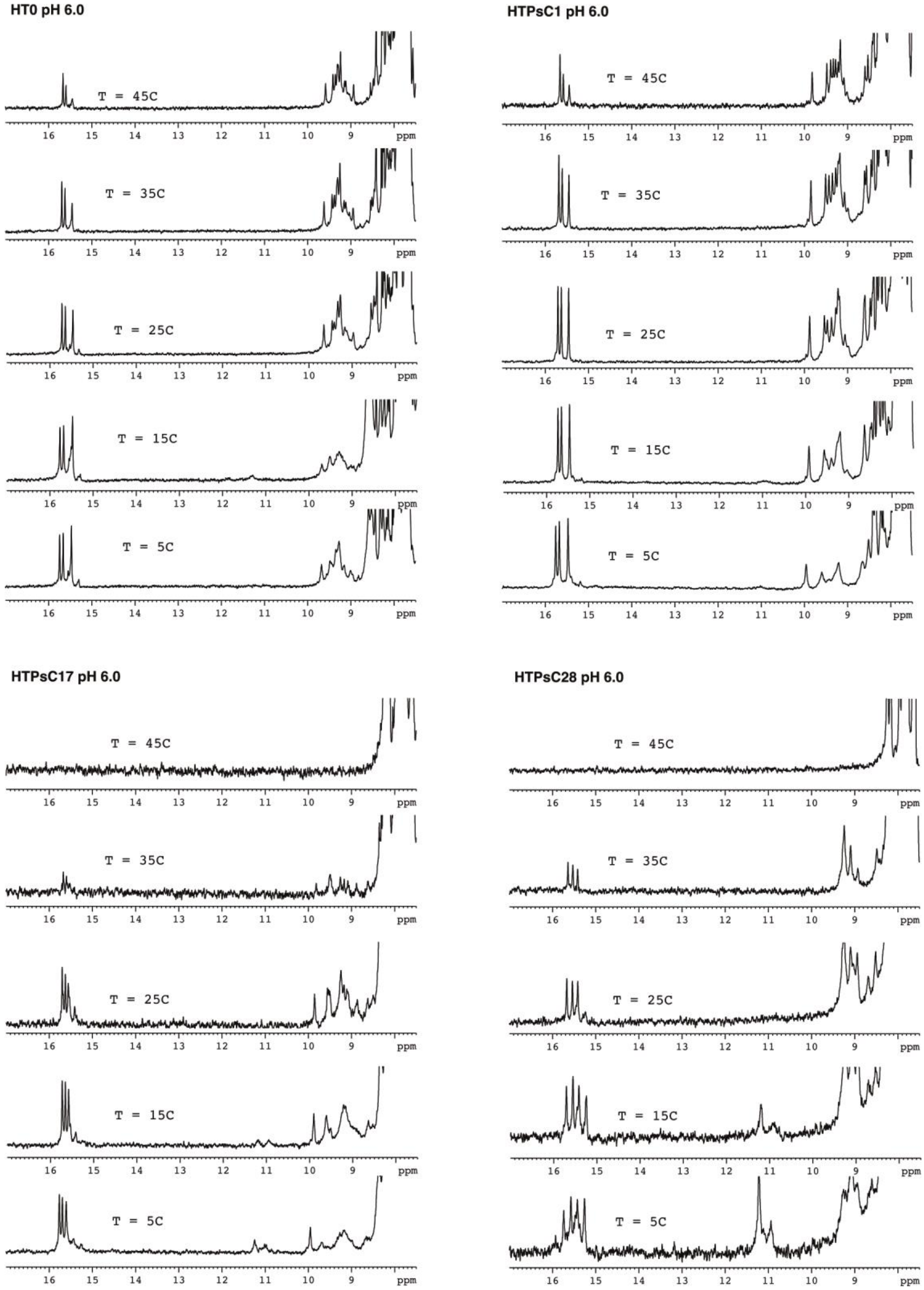

Figure S16. Series of 1D ^{1}H-NMR spectra at different temperature and pH 6 of **HT-0**, **HT-psC1**, **HT-psC17** and **HT-psC28**. Experimental conditions: 25 mM phosphate buffer, [oligonucleotide] = 0.1 mM.

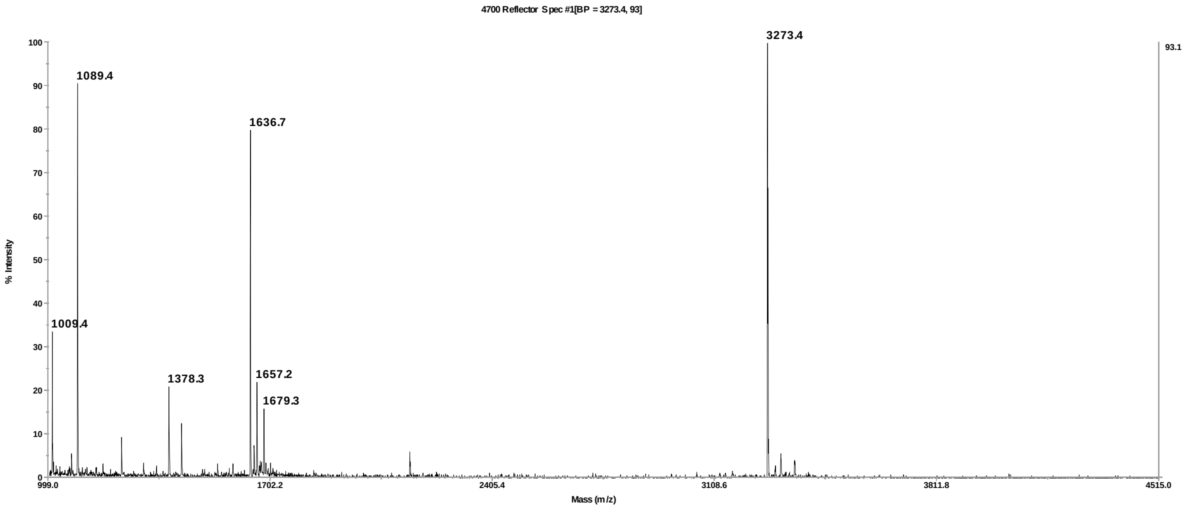

Figure S17. MALDI-TOF spectrum of **CC0**.

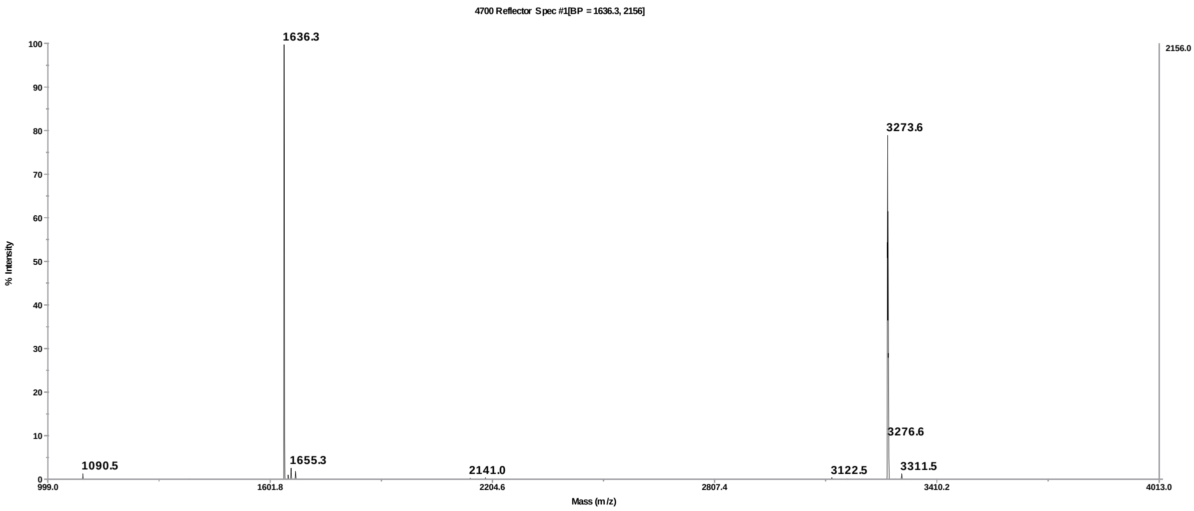

Figure S18. MALDI-TOF spectrum of **CC9**.

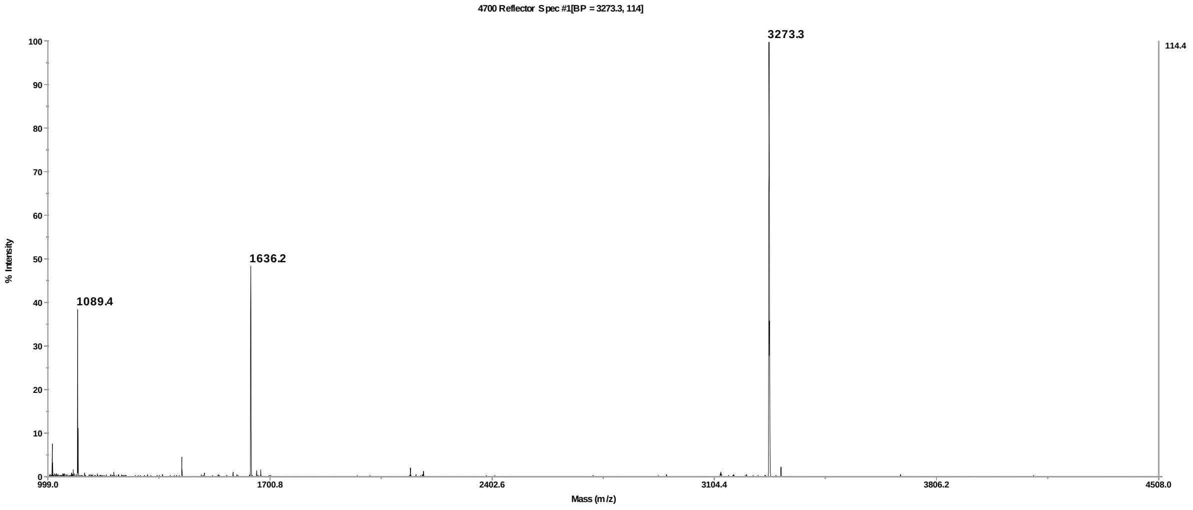

Figure S19. MALDI-TOF spectrum of **CC8**.

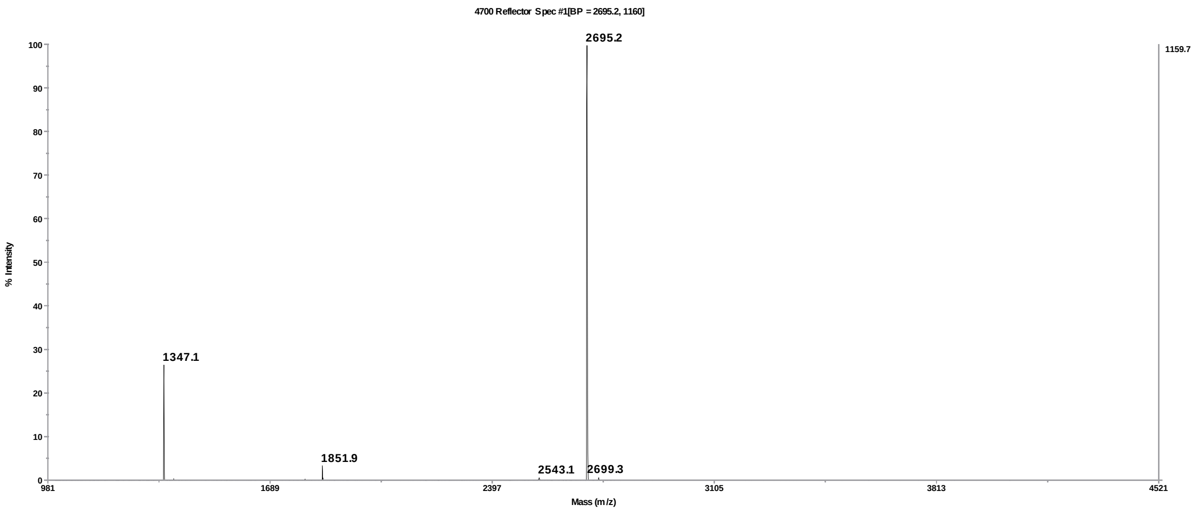

Figure S20. MALDI-TOF spectrum of **C7**.

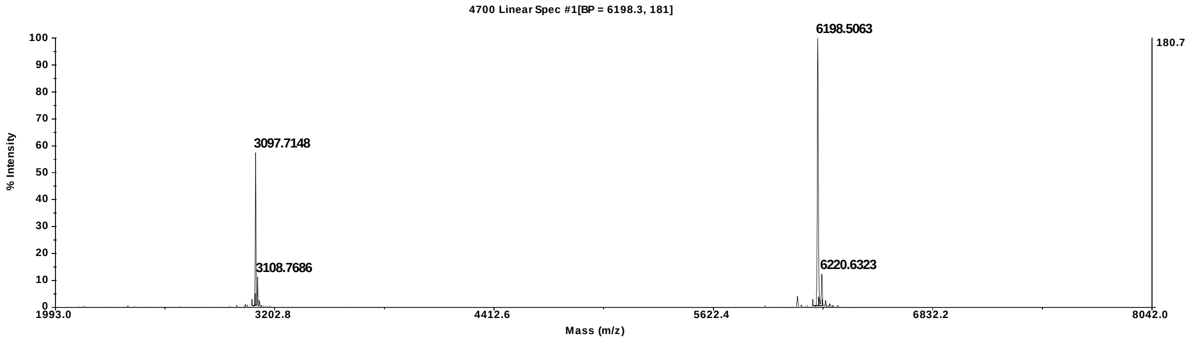

Figure S21. MALDI-TOF spectrum of **HT0**.

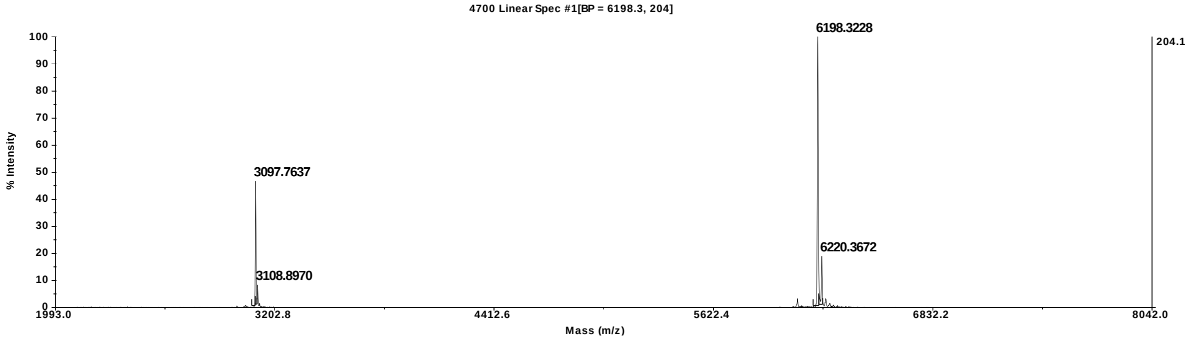

Figure S22. MALDI-TOF spectrum of **HT-psC1**.

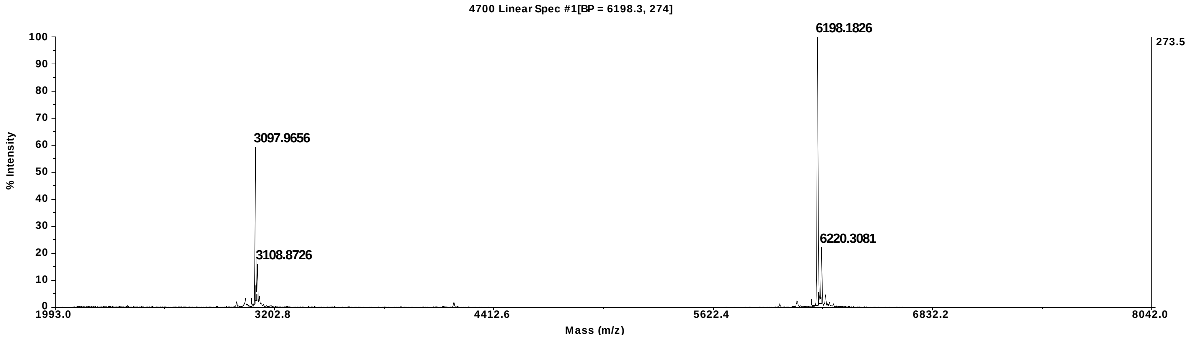

Figure S23. MALDI-TOF spectrum of **HT-psC17**.

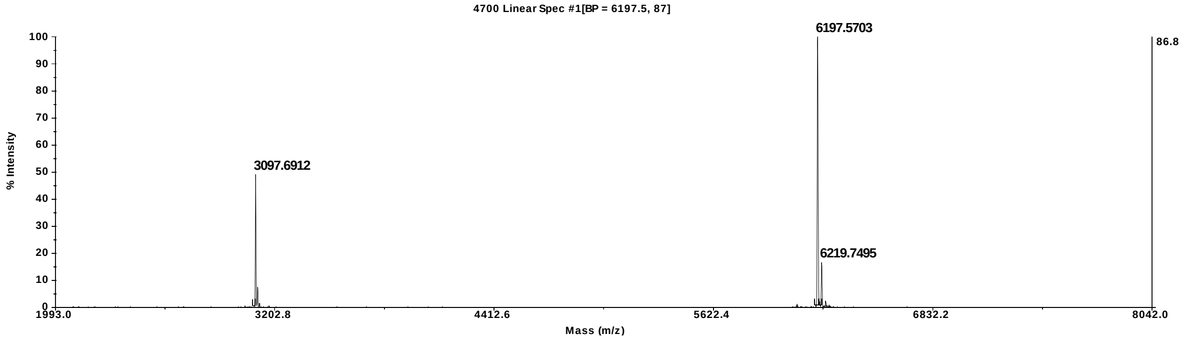

Figure S24. MALDI-TOF spectrum of **HT-psC28**.

3. Supplementary Tables.

Table S1. Oligonucleotides synthesis results.

Sequence	ε (mL·µmol^{-1}·cm^{-1})	Crude Yield %	HPLC Purity %	Purified Yield %	Theoretical Mass	MS-MALDI-TOF m/z
CC0	92,5	88	83	27	3274,2	3274,2
C7	78,1	68	74	19	2695,8	2695,2
CC8	92,5	79	62	31	3274,2	3273,3
CC9	92,5	74	72	34	3274,2	3273,6
HT-psC1	185,9	60	89	8	6200,1	6198,3
HT-psC17	185,9	77	83	15	6200,1	6198,2
HT-psC28	185,9	62	84	11	6200,1	6197,7

Table S2. Chemical shifts of non-exchangeable protons of **CC0**, pH 4, T=5°C

Residue	H6/H8	H5/Me	H1'	H2'	H2"	H3'	H4'	H5'/H5"
T1	7.61	1.77	6.21	2.31	2.43	4.73	4.12	3.69
C2/C8	7.89/7.85	5.89	6.37	1.94/1.86	2.38	4.74	4.19	3.98
C3	7.40	5.76	5.90	1.36	2.00	-	-	-
G4	8.12	-	5.74	2.76	2.42	-	-	-
T5	7.43	1.59	5.93	1.91	2.19	4.72	4.29	3.86
T6	7.77	1.90	6.27	2.32	2.424	4.58	4.30	
T7	7.63	1.80	6.29	2.42	1.89	-	-	-
C9	7.39	5.72	5.89	1.42	2.09	-	-	-
G10	8.09	-	5.75	2.73	2.40	-	-	-
T11	7.48	1.58	5.89	1.96	2.08	4.32		3.89

Table S3. Chemical shifts of exchangeable protons of **CC0**, corresponding to the head-to-head and head-to-tail species, pH 4, T=5°C

	Head-to-head			Head-to-tail			
Residue	H1/H3	H42/H22	H41/H21	Residue	H1/H3	H42/H22	H41/H21
T1	n.o.	-	-	T1	n.o.	-	-
C2/C8	15.47	9.22	8.30	C2/C8	15.47	9.22	8.30
C3 (C3:C3$^+$)	15.25	9.47	7.88	C3 (C3:C9$^+$)	15.23	9.43	8.14
G4	10.47	8.37	6.14	G4	10.34	8.43	6.24
T5	n.o.	-	-	T5	n.o.	-	-
T6	n.o.	-	-	T6	n.o.	-	-
T7	11.64	-	-	T7	11.70	-	-
C9 (C9:C9$^+$)	15.23	9.29	7.96	C9 (C3:C9$^+$)	15.23	-	-
G10	10.30	8.40	6.22	G10	10.36	8.36	6.22
T11	n.o.	-	-	T11	n.o.	-	-

Table S4. Chemical shifts of **CC9**, corresponding to the head-to-tail species, pH 7, T=5°C

Residue	H1/H3	H42/H22	H41/H21	H6/H8	H5/Me	H1’	H2’	H2”	H3’
T1/T7	n.o.	-	-	7.62	1.79	-	-	-	n.a.
C2 (C2:C8^{+})	15.56	9.61	8.09	7.84	5.83	6.32	2.06	2.45	4.71
C3	-	8.83	7.01	7.83	5.54	5.91	1.43	1.96	4.53
G4	10.39	8.35	6.00	8.03	-	5.77	2.72	2.38	n.a.
T5/T11	n.o.	-	-	7.50	1.59	5.84	2.00		4.34
T6	n.o.	-	-	7.38	1.61	5.95	2.05	2.34	4.57
C8 (C2:C8^{+})	15.56	8.93	7.54	7.66	5.78	6.34	1.68	2.37	n.a.
psC9	14.05	8.67	6.10	7.03	-	5.90	1.41	1.95	n.a.
G10	9.70	8.34	6.49	8.10	-	5.70	2.74	2.38	n.a.

Table S5. Chemical shifts of **CC8**, corresponding to the head-to-head species with C9-C2-psC8-C3 stacking order, pH 5, T=1°C

Residue	H1/H3	H42/H22	H41/H21	H6/H8	H5/Me	H1’	H2’	H2”	H3’
T1	11.44	-	-	7.65	1.79	6.27	2.39	2.21	4.75
C2 (C2:C2^{+})	15.48	9.21	8.37	7.91	5.97	6.35	1.72	2.43	4.76
C3	15.26	9.51	7.86	7.39	5.86	7.39	1.37	2.07	4.45
G4	10.15	8.26	6.37	8.12	-	5.75	2.77	2.41	4.95
T5	10.35	-	-	7.43	1.62	5.97	1.92	2.19	4.79
T6	11.05	-	-	7.63	1.83	6.32	n.a.	n.a.	n.a.
T7	11.51	-	-	7.83	1.93	6.37	2.33	2.53	4.90
psC8 (psC8:psC8^{+})	15.90	9.34	7.60	7.47	-	6.40	1.96	2.33	n.a.
C9 (C9:C9^{+})	15.43	9.57	8.04	7.43	5.76	5.85	1.63	2.08	n.a.
G10	10.10	8.39	6.44	8.12	-	5.75	2.77	2.41	4.95
T11	10.61	-	-	7.48	1.50	5.91	1.99	2.11	4.34

Table S6. Chemical shifts of **C7**, corresponding to the head-to-tail species, pH 5, T=1°C

Residue	H1/H3	H42/H22	H41/H21	H6/H8	H5/Me	H1’	H2’	H2”	H3’
T1	11.41	-	-	7.65	1.75	6.36	2.39	2.52	n.a.
C2 (C2:psC7^{+})	-	9.20	6.90	7.24	6.06	6.14	0.84	2.12	4.63
G3/G8	10.11	8.55	6.05	8.18	-	5.78	2.79	2.46	4.95
T4/T9	n.a.	-	-	7.47	1.59	5.90	1.94	2.17	4.64
T5	n.a.	-	-	7.63	1.77	6.29	2.14	2.44	n.a.
T6	n.a.	-	-	7.69	1.82	6.43	2.33	2.52	4.77
psC7 (C2:psC7^{+})	14.71	9.00	6.15	7.18	-	6.14	0.72	1.94	n.a.

Table S7. Chemical shifts of **C7**, corresponding to the head-to-head species, pH 4, T=1°C

Residue	H1/H3	H42/H22	H41/H21	H6/H8	H5/Me	H1'	H2'	H2"	H3'
T1	n.a.	-	-	7.69	1.72	6.31	2.47		4.41
C2 (C2:C2$^+$)	15.29	9.44	7.29	7.38	5.96	6.17	0.74	1.99	4.64
G3	10.30	8.72	5.75	8.25	-	5.80	2.86	2.53	4.32
T4	n.a.	-	-	7.48	1.61	5.91	1.95	2.18	4.63
T5	n.a.	-	-	7.64	1.76	6.33	2.14	2.43	4.41
T6	11.65	-	-	7.77	1.89	6.39	2.39	2.50	n.a.
psC7 (psC7:psC7$^+$)	15.65	9.62	7.02	7.29	-	6.39	0.99	2.17	n.a.
G8	10.79	8.67	5.91	8.21	-	5.72	2.78	2.47	n.a.
T9	n.a.	-	-	7.46	1.60	5.91	1.95	2.18	4.63

Table S8: Experimental constraints and calculation statistics of **CC9**.

Experimental constraints and calculation statistics of **CC9**.		
Experimental distance constraints		
Total number	128	
Intra-residue	66	
Sequential	36	
Range > 1	26	
Intra-subunit	108	
Inter-subunit	20	
RMSD	(Å)	
All well-defined bases$^+$	0.5 ± 0.2	
All well-defined heavy atoms$^+$	0.8 ± 0.3	
Backbone	1.8 ± 0.5	
All heavy atoms	2.5 ± 0.7	
Residual violations	Average	Range
Sum of violation (Å)	7.2	6.3 .. 7.9
Max. violation (Å)	0.78	0.34 .. 0.94
NOE energy (kcal/mol)	35	24 .. 41
Total energy (kcal/mol)	-1615	-1490 .. -1739

$^+$All except unpaired thymines.
